# Nanopores Reveal the Stoichiometry of Single Oligoadenylates Produced by Type III CRISPR-Cas

**DOI:** 10.1021/acsnano.3c11769

**Published:** 2024-06-14

**Authors:** David Fuentenebro Navas, Jurre A. Steens, Carlos de Lannoy, Ben Noordijk, Michael Pfeffer, Dick de Ridder, Raymond H.J. Staals, Sonja Schmid

**Affiliations:** † Laboratory of Biophysics, Wageningen University and Research, Stippeneng 4, 6708WE Wageningen, The Netherlands; ‡ Laboratory of Microbiology, Wageningen University and Research, Stippeneng 4, 6708WE Wageningen, The Netherlands; § Bioinformatics Group, Wageningen University and Research, Droevendaalsesteeg 1, 6708PB Wageningen, The Netherlands; ∥ Department of Bionanoscience, Delft University of Technology, Van der Maasweg 9, 2629HZ Delft, The Netherlands; ⊥ Department of Chemistry, University of Basel, Mattenstrasse 22, 4058 Basel, Switzerland

**Keywords:** nanopore, CRISPR-Cas, type III, neural network, single-molecule detection, second messenger

## Abstract

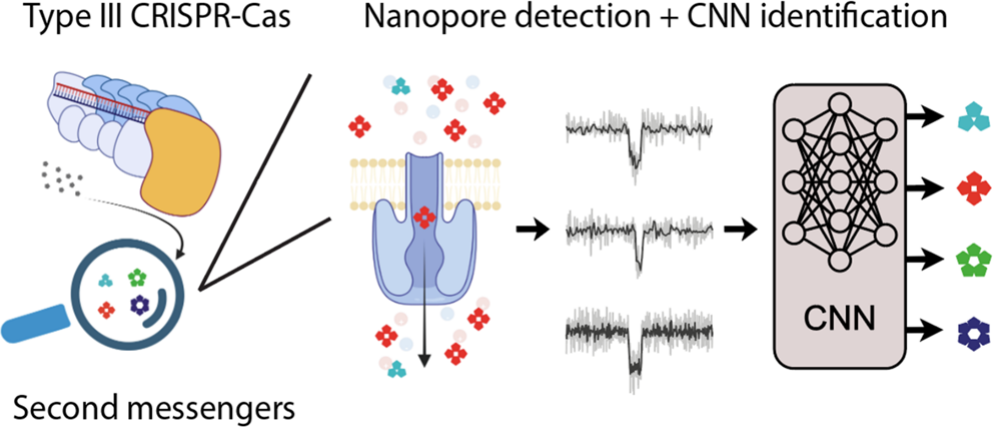

Cyclic oligoadenylates (cOAs) are small second messenger molecules produced by the type III CRISPR-Cas system as part of the prokaryotic immune response. The role of cOAs is to allosterically activate downstream effector proteins that induce dormancy or cell death, and thus abort viral spread through the population. Interestingly, different type III systems have been reported to utilize different cOA stoichiometries (with 3 to 6 adenylate monophosphates). However, so far, their characterization has only been possible in bulk and with sophisticated equipment, while a portable assay with single-molecule resolution has been lacking. Here, we demonstrate the label-free detection of single cOA molecules using a simple protein nanopore assay. It sensitively identifies the stoichiometry of individual cOA molecules and their mixtures from synthetic and enzymatic origin. To achieve this, we trained a convolutional neural network (CNN) and validated it with a series of experiments on mono- and polydisperse cOA samples. Ultimately, we determined the stoichiometric composition of cOAs produced enzymatically by the CRISPR type III-A and III-B variants of *Thermus thermophilus* and confirmed the results by liquid chromatography–mass spectroscopy (LC-MS). Interestingly, both variants produce cOAs of nearly identical composition (within experimental uncertainties), and we discuss the biological implications of this finding. The presented nanopore-CNN workflow with single cOA resolution can be adapted to many other signaling molecules (including eukaryotic ones), and it may be integrated into portable handheld devices with potential point-of-care applications.

Second messenger molecules and other small metabolites serve a wide variety of essential signaling, activation, and regulation purposes in the biological cell, such as spatial and temporal regulation of cellular responses, signal transduction between cell membrane and nucleus, and neuro-transmission.^1^ However, due to their small size, they are particularly difficult to detect, quantify, and study. In the present work, we focus on cyclic oligoadenylate molecules (cOAs) which play a crucial role in the type III CRISPR-Cas adaptive immune system of prokaryotes.^2,3^ This immune system evolved among bacteria and archaea to combat invading plasmids, bacteriophages, and other mobile genetic elements (MGEs).^4−6^ It works by storing short DNA sequences of the encountered MGEs in an array of clustered, regularly interspaced short palindromic repeats, the CRISPR array.^7^ During subsequent infections, the CRISPR array is transcribed, processed into short CRISPR RNAs (crRNAs, or guide RNAs), and incorporated into a single CRISPR-associated (Cas) protein or into multisubunit protein complexes, with different modes of action (classified in several classes and types).^7,8^ The crRNA-protein complexes then bind and degrade invading complementary MGEs.

The cOAs are second messengers produced by the type III CRISPR-Cas complex (Figure 1A). This ribonucleoprotein complex is endowed with three catalytic activities: sequence-specific RNAase activity by the Cas7 subunits, nonspecific ssDNA cleavage by the HD domain of Cas10, and the ATP-cyclase activity of the palm domain of Cas10 responsible for generating the cOAs.^3,9^ The stoichiometry of these cOA molecules can vary between different hosts and CRISPR type III subtypes, but they typically contain three to six adenosine monophosphates (AMP) in a ring structure (cA_3_–cA_6_, Figure 1A).^2,10,11^ The cOAs of the type III CRISPR response were found to activate particular families of downstream effector proteins containing an appropriate sensory domain, such as CRISPR-associated Rossman fold (CARF) and (SAVED) second messenger oligonucleotide or dinucleotide synthetase (SMODS)-associated and fused to various effector domains. The cOA-binding domain of these proteins is often fused to other different domains with a wide variety of catalytic activities, such as (ribo-)nucleases and proteases.^8,12^ Activation of these enzymes by the cOAs results in a “secondary line of defense” by type III systems, leading to the degradation of essential host biomolecules that induce either cellular dormancy or even cell death, preventing viral (or other MGE) propagation through the population.^13,14^

**Figure 1:**
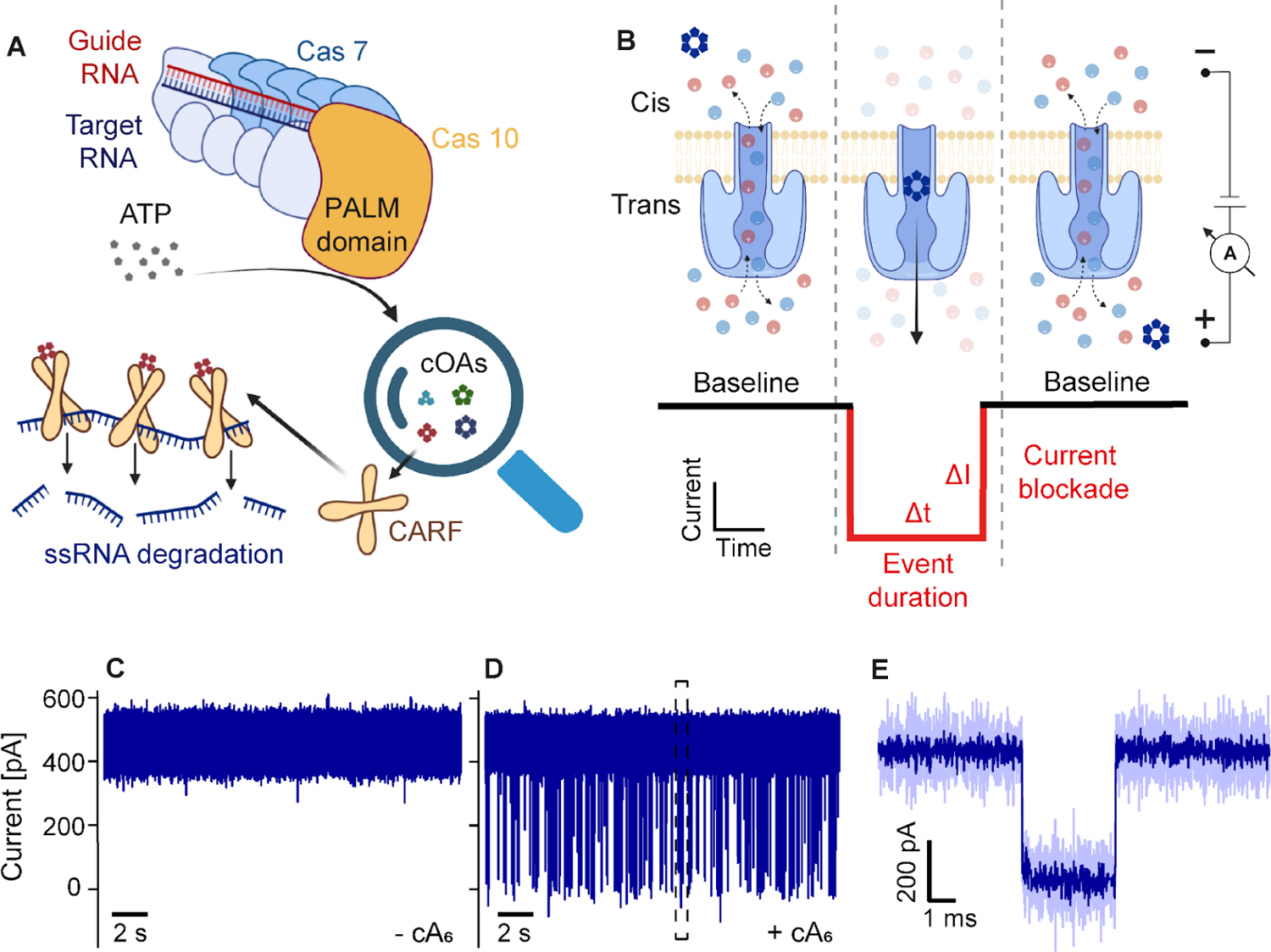
Nanopore detection of single CRISPR second messenger molecules. (A) Schematic depiction of a type III CRISPR-Cas complex. Target RNA binding activates the cyclase activity of the palm domain of Cas10 for cOA synthesis from ATP molecules. The cOA molecules activate CARF proteins, such as the nonspecific ribonuclease Csx1. (B) Schematic arrangement of the α-hemolysin (α-HL) nanopore experiment. Upon voltage application, an ionic current flows through the pore. A cOA translocation, driven by electrophoresis, is observed as a resistive pulse with current blockade Δ*I* and duration Δ*t*. We refer to the depicted pore arrangement as a “trans-inserted” α-HL, where the vestibule is on the trans side. (C–E) α-HL recordings at +200 mV in a 3 M KCl buffer, obtained as illustrated in (B). (C) Baseline trace measured without an analyte. (D) α-HL recording after the addition of 10 μM cA_6_ to the cis compartment. Both (C) and (D) were recorded in triplicate, using three individual pore proteins (Figure S6). (E) Single cA_6_ translocation event extracted from the trace in (D), as indicated by the dashed rectangle. The dark blue line represents filtered data, and the light blue line represents measured raw data (see the Methods Section).

As the diverse catalytic functions that are activated by cOAs are only just being unveiled, several questions remain unsolved. In particular: do the Cas10 homologues all produce monodisperse cOAs, or rather a polydisperse distribution? In addition, it has been reported that several distinct type III CRISPR systems (including different CARF and SAVED proteins) can be found within one host, despite using the same CRISPR array.^15−17^ For example, in *Thermus thermophilus* HB8, considered here, the genome encodes two different CRISPR-Cas type III systems, termed III-A and III-B. This is unexpected, and raises the question: what is the evolutionary benefit of having multiple distinct systems for a given task? One possible reason for the co-occurrence of multiple type III subtypes in one host is that they may each produce a unique subset of cOA stoichiometries, thus providing a regulatory benefit by activating distinct effector proteins in a fine-tuned, cOA-stoichiometry-dependent way. To test this hypothesis and elucidate cOA-dependent regulation mechanisms, a simple and rapid method to directly detect small amounts of enzymatically produced cOAs, and even quantify their stoichiometric composition with single-molecule resolution would be instrumental.

Here, we demonstrate the label-free detection of single cOA molecules and their stoichiometries using nanopore experiments, where a pore protein embedded in a free-standing lipid bilayer acts as a sensor for single cOA molecules (Figure 1B). An applied positive voltage drives the negatively charged cOA molecules through the nanopore by electrophoresis. While translocating, the cOA partially blocks the ionic through-pore current, resulting in a characteristic current blockade signal (cf. resistive pulse sensing).^18^ Nanopores are by definition single-molecule sensors due to their small size, which we chose here to be comparable to the cOA molecule. More generally, nanopore technology is best known for commercial devices offering inexpensive and portable DNA sequencing with long-reads that are revolutionizing the life sciences.^19,20^ The sensitivity of protein nanopores is surprising: even single enantiomers (chiral variants) in small-molecular racemates can be distinguished.^21,22^ In comparison to established ensemble techniques, a nanopore-based cOA detector with single-molecule resolution would bring many practical and fundamental advantages. First, nanopore detectors are simple, fast, affordable, and highly parallelizable as demonstrated by the commercialized DNA sequencers,^19^ and they can be used on the bench or as portable devices in the field,^19,23^ whereas current alternatives, such as mass spectrometers are larger, and generally require trained staff in dedicated research facilities. Moreover, the currently established techniques suffer from fundamental limitations, such as a narrow dynamic range of mass spectrometry,^24^ because they rely on averaging over large ensembles of molecules. As such, the inability of established techniques to detect single messenger molecules prohibits the resolution of rare but decisive species within a majority of other molecules. By contrast, nanopore technology offers the necessary single-molecule resolution to overcome these fundamental limitations, as previously demonstrated.^25−29^

Neural networks play a crucial role in nanopore signal processing.^30−32^ An important advantage of neural networks is their ability to implicitly extract features from the data provided during training, in contrast to other machine learning approaches that require the manual definition of informative features (*e.g*., hidden Markov models). Neural networks are, therefore, able to learn and combine more subtle characteristics, such as the shapes of blockade events and their current fluctuations.^33,34^ For nanopore signal processing, this enabled improved quantitative analyses.^30^ In this study, we use a convolutional neural network (CNN) to quantitatively infer the stoichiometric composition of cOA mixtures, including samples from enzymatic origin.

Here, we demonstrate the detection of cOA second messengers with single-molecule resolution, using a sensitive nanopore assay. We compare a range of synthetic cOAs with known monodisperse stoichiometries of three to six adenosine monophosphate (AMP) monomers (subsequently: cA_3_ to cA_6_). Using this calibration data as a training set for our CNN, we turn to cOA mixtures of known polydisperse composition and validate the capacity of the CNN to quantify the correct ratio of stoichiometries involved. We then use this label-free cOA identification pipeline to study enzymatic cOA samples. Specifically, we identify the stoichiometric composition of cOAs produced by different type III-A and III-B complexes and compare them with their CARF activation capability. Liquid chromatography–mass spectroscopy (LC-MS) further confirms our nanopore results. Lastly, we discuss the implications of our results on the hypothesized evolutionary benefit of multiple type III CRISPR systems (possibly producing varied cOA stoichiometries) in one prokaryotic host. Altogether, our label-free cOA identification assay has proven its capacity to identify small second messengers with single-molecule resolution—even from enzymatic mixtures—and also their stoichiometry. This makes nanopore detection a promising tool for future metabolic research of CRISPR-Cas and beyond, where fast, label-free, and quantitative readouts matter.

## Results and Discussion

### Detecting Single Cyclic Oligoadenylates with Protein Nanopores

Figure 1 shows the capacity of the α-hemolysin (α-HL) pore protein to detect single cOA molecules. In contrast to the clean α-HL baseline measured in the absence of cOA molecules (Figure 1C), characteristic resistive pulses appear after cOA addition to the “cis” side of the pore (see definition in Figure 1B, and data in Figure 1D), each representing a single cOA translocation (Figure 1E). As expected, the cOA event rate is concentration-dependent (Figure S1), and the event duration decreases with increasing voltage (Figure S2 and Table S1), indicating that cis-to-trans translocations take place (rather than cis-to-cis escapes). This behavior is expected for substantially charged molecules, like the poly nucleic cOAs, for which the electrophoretic driving force dominates over other contributions (*e.g*., electro-osmosis).^35^ The current blockade Δ*I* and event duration Δ*t* depend on the specific analyte and measurement conditions. For cOAs with a stoichiometry of six AMP subunits (cA_6_), we find mean values and standard deviations of Δ*I* = 400 ± 30 pA and Δ*t* = 315 ± 26 μs, measured at +200 mV in 3 M KCl (Figure 1D,E and Table S1).

For the single-molecule resolution of these small cOA molecules, the choice of the nanopore and precise experimental conditions is crucial, as several factors affect the sensing performance. For example, while the MspA nanopore with its much pointier constriction site provides better spatial resolution than α-HL in DNA sequencing applications,^36,37^ it barely resolved cOA translocations (Figures S3 and S4), which limits its utility for our application in small-molecule sensing. In contrast, the α-HL with its long narrow stem yields single-molecule observations that are well resolved in time, even for these small cOA molecules. In addition, we found that the translocations through a trans-inserted α-HL (illustrated in Figure 1B) were more uniform than the translocations with a cis-inserted pore (Figure S5). Similar findings have previously been attributed to less heterogeneous excursions and interactions in the wide pore lumen of α-HL.^38^ Overall, a trans-inserted α-HL in 3 M KCl and +200 mV bias provided an optimal signal-to-noise ratio to resolve single cA_6_ molecules.

### Nanopore Event Durations Reveal the Stoichiometry of cOA Molecules

**Figure 2:**
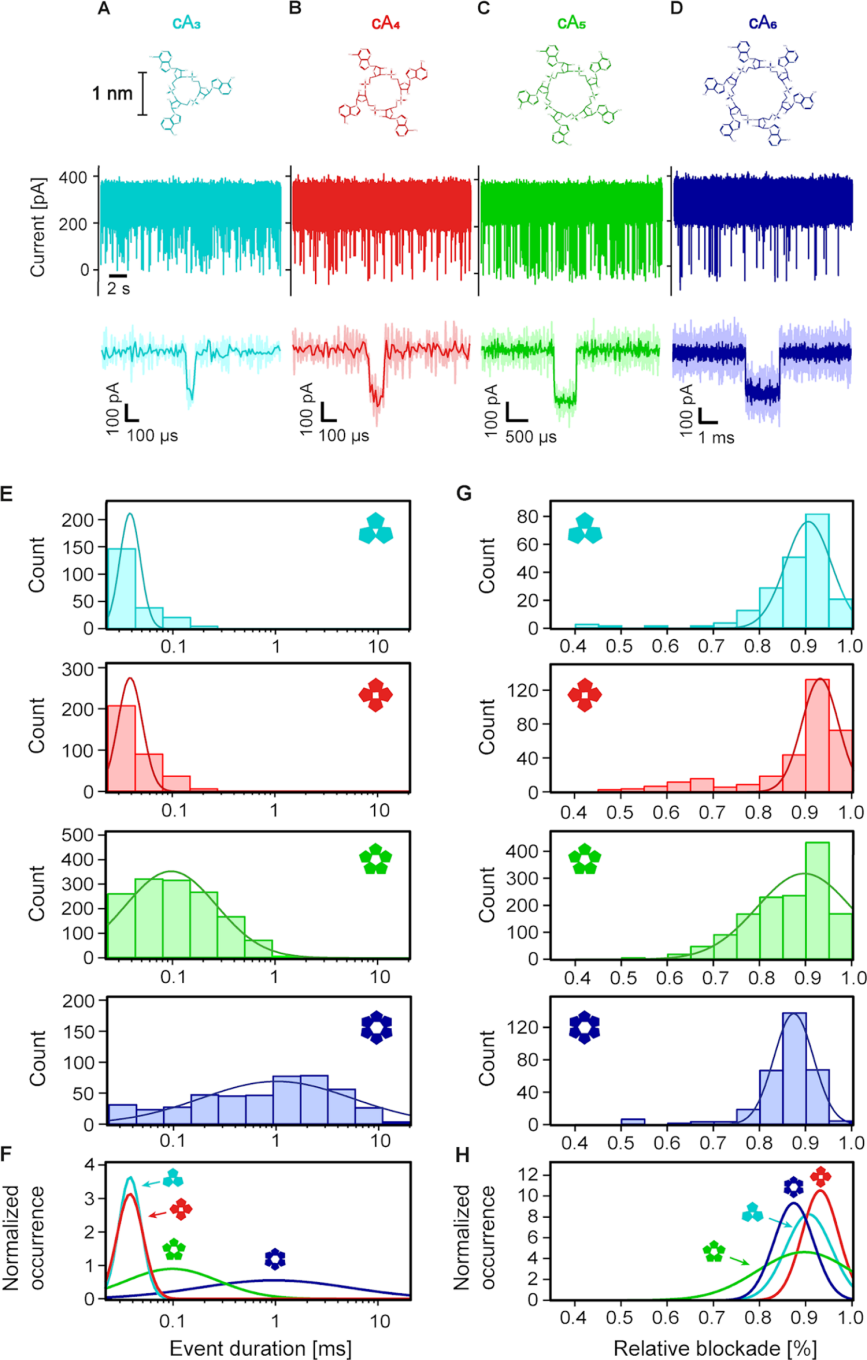
Nanopore recordings reveal the stoichiometry of cyclic oligoadenylates. (A–D) Nanopore recordings showing the label-free detection of single cOA molecules with different stoichiometries: cA_3_, cA_4_, cA_5_, and cA_6_, respectively. Top: molecular structure of the cOAs with 3–6 AMP monomers as indicated (same scale bar for (A–D)). Middle: nanopore current trace with short blockade events indicating cOA translocations, obtained at +120 mV with 10 μM of the respective cOAs (same scale bar and axis for (A–D)). Bottom: zoom-in on a single translocation event of the respective cOA. (E) Event duration histograms for the four cOAs with log-normal fits to the data. (F) Overlay of all distributions in (E), with normalized integrals. (G) Relative blockade histograms for the four cOA data sets in (E) with normal fits to the data. (H) Overlay of the distributions in (G) with normalized integrals. All nanopore data were recorded in triplicate, with individual pores (Figure S6).

Encouraged by the successful label-free single-molecule detection of cA_6_, we probed the sensitivity of our assay to detect even smaller cOAs: cA_3_, cA_4_, and cA_5_ depicted in Figure 2A–D. Biologically, this is highly relevant, since type III CRISPR-Cas complexes have been reported to produce cOAs with varying stoichiometries.^39^ Experimentally, however, it has been impossible up to now to resolve these differences among *single* cOA molecules, given their small, cyclized structure (Figure S7 shows a three-dimensional (3D) representation). Gratifyingly, with the presented nanopore assay, all cOAs down to cA_3_ can be resolved at +120 mV, as evident from the current traces and zoomed-in events shown in Figure 2A–D. The measured event durations scale with the cOA size, as the larger cOAs have a reduced translocation probability through α**-**HL’s 1.4 nm constriction site (Figures 2E,F, S8, S9 and Table S1). Interestingly, however, cA_3_ to cA_6_ all cause similar current blockades, *i.e*., the larger cOAs do not measurably block more ionic current (Figures 2G,H, S8 and S9), likely due to local positioning inside the nanopore and 3D folding effects (cf. Figure S7). To distinguish cOA stoichiometries, we therefore focus on the distinct event durations. Their clear trend can serve as a proxy for cOA identification, while it is also evident that cA_3_ and cA_4_ cannot yet be distinguished based on the overlapping event durations alone (Figure 2E, bottom). We therefore turned to neural networks, which have the ability to recognize more subtle patterns in the nanopore translocation events and allow the identification of cOA stoichiometries at the single-event level.

### Neural Networks Can Infer the Stoichiometry of Single cOA Molecules

**Figure 3:**
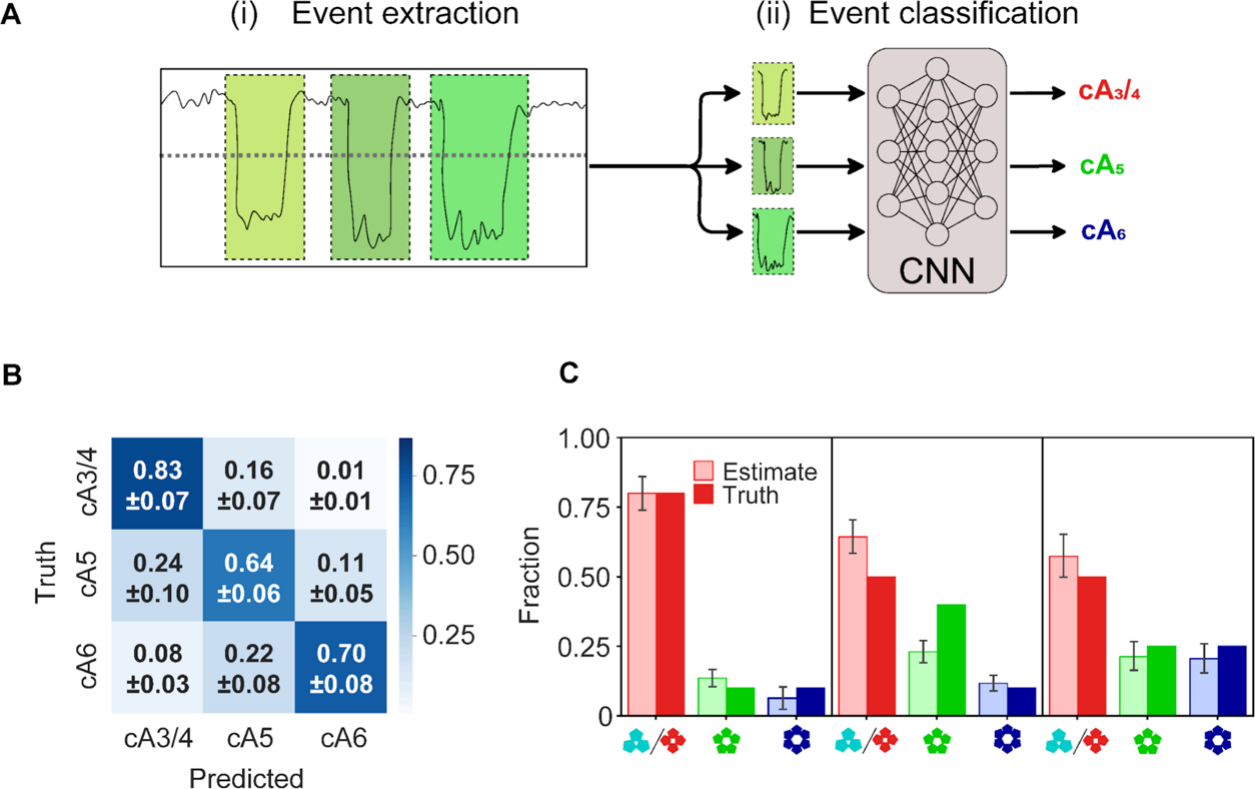
Neural network workflow and validation of the cOA stoichiometry inference from nanopore recordings. (A) The cOA classification pipeline consists of (i) extraction of the cOA translocation events, (ii) cOA classification per event using a convolutional neural network (CNN). See the Methods Section for details and code. (B) Confusion matrix showing the performance for the single-event classification of 2058 cOA translocation events (not included in the training set). Fractions denote relative prediction frequencies per ground truth class (mean ± standard deviation over 10 cross-validation folds). (C) cOA stoichiometry distribution analysis: comparison of the experimental input (dark-colored) *vs* the inferred output (light-colored) obtained for cOA mixtures of known composition, as indicated (percentages from left to right: 80:10:10, 50:40:10, and 50:25:25). Error bars denote 95% prediction intervals obtained by 10-fold cross-validation, which express uncertainty introduced by data variability given this CNN architecture and fitting procedure.

Neural networks have proven very useful in nanopore signal interpretation,^40^ thanks to their ability to recognize features beyond the mere event duration and current blockade discussed above. Hence, to differentiate between nanopore events caused by cOAs of different stoichiometries, we trained a convolutional neural network (CNN) for single-event classification (Figure 3A). The training data consisted of cOA events obtained from monodisperse cOA samples with known stoichiometry (see the Methods Section), and the data used for model evaluation was obtained from different experiments than the training data, to achieve valid accuracy estimates. Indeed, our CNN outperforms a more conventional machine learning approach (a k-nearest neighbor classifier considering only event duration and current blockade, Figure S10), which suggests that the CNN recognizes additional signal properties, such as event shape and current fluctuations. Nevertheless, the events of the two smallest cOAs (*i.e*., cA_3_ and cA_4_, with very similar duration and current blockades) are not well separated by the CNN. We note that this can likely be solved, in the future, using alternative (possibly engineered) protein nanopores, capable of distinguishing both stoichiometries. For this study, however, we move on with a joint class of cA_3/4_. Using this approach, the CNN correctly identified 83, 64, and 70% of (unseen) monodisperse cA_3/4_, cA_5_, and cA_6_ events, respectively (Figure 3B). Erroneous classifications occur mainly between adjacent stoichiometries (*e.g*., cA_6_ misclassified as cA_5_, or cA_5_ misclassified as cA_6_ or cA_3/4_), and mainly toward lower stoichiometries (*e.g*., cA_5_ misclassified as cA_3/4_, rather than cA_6_). The latter reflects the event duration distributions and their overlap (Figure 2): cA_5_ and cA_6_ events misclassified as cA_3/4_ are marked by short event durations (Figure S11). Altogether, 73% of all unseen events in monodisperse samples are correctly identified by the CNN.

### Quantifying Polydisperse cOA Mixtures and CNN Validation

We next assessed the ability of the trained CNN to estimate the stoichiometric composition of polydisperse samples. We prepared three cOA mixtures with known composition and acquired nanopore recordings of each one. Figure 3C shows the true and inferred stoichiometry fractions, with prediction intervals obtained by 10-fold cross-validation (see the Methods Section). In qualitative terms, the relative abundances (high *vs* low) are correctly identified in all cases, down to the smallest tested fraction of 10%. Quantitatively, the cA_3/4_ population is partially overestimated, while the cA_5_ population is sometimes underestimated, which is consistent with our results for the monodisperse cOAs (previous section). For the 50:40:10 mixture (Figure 3C, middle), the deviation is larger than the model’s prediction interval, indicating additional imperfections beyond the training and test data variability.

Importantly, all ground truth differences in population size were correctly identified by the CNN, which inferred significantly different populations in these cases (*t* test, *p* ≪ 0.01, Table S2). Similarly, identical ground truth populations are also inferred to be identical within the experimental uncertainties—with the exception of the smallest populations of 10%. This indicates the resolution limit of the CNN classification procedure for population sizes, which we (conservatively) estimate to be 15%. Individual uncertainties for the cOA identification procedure can be estimated as 17% for cA_3,4_, 36% for cA_5_, and 30% for cA_6_, based on the confusion matrix (1—diagonal value in Figure 3B). Equipped with this nanopore-CNN workflow with validated accuracies and uncertainty estimates, we moved on to study cOA mixtures of enzymatic origin.

### Stoichiometry of cOAs Produced by Type III-A and III-B CRISPR-Cas Complexes

**Figure 4:**
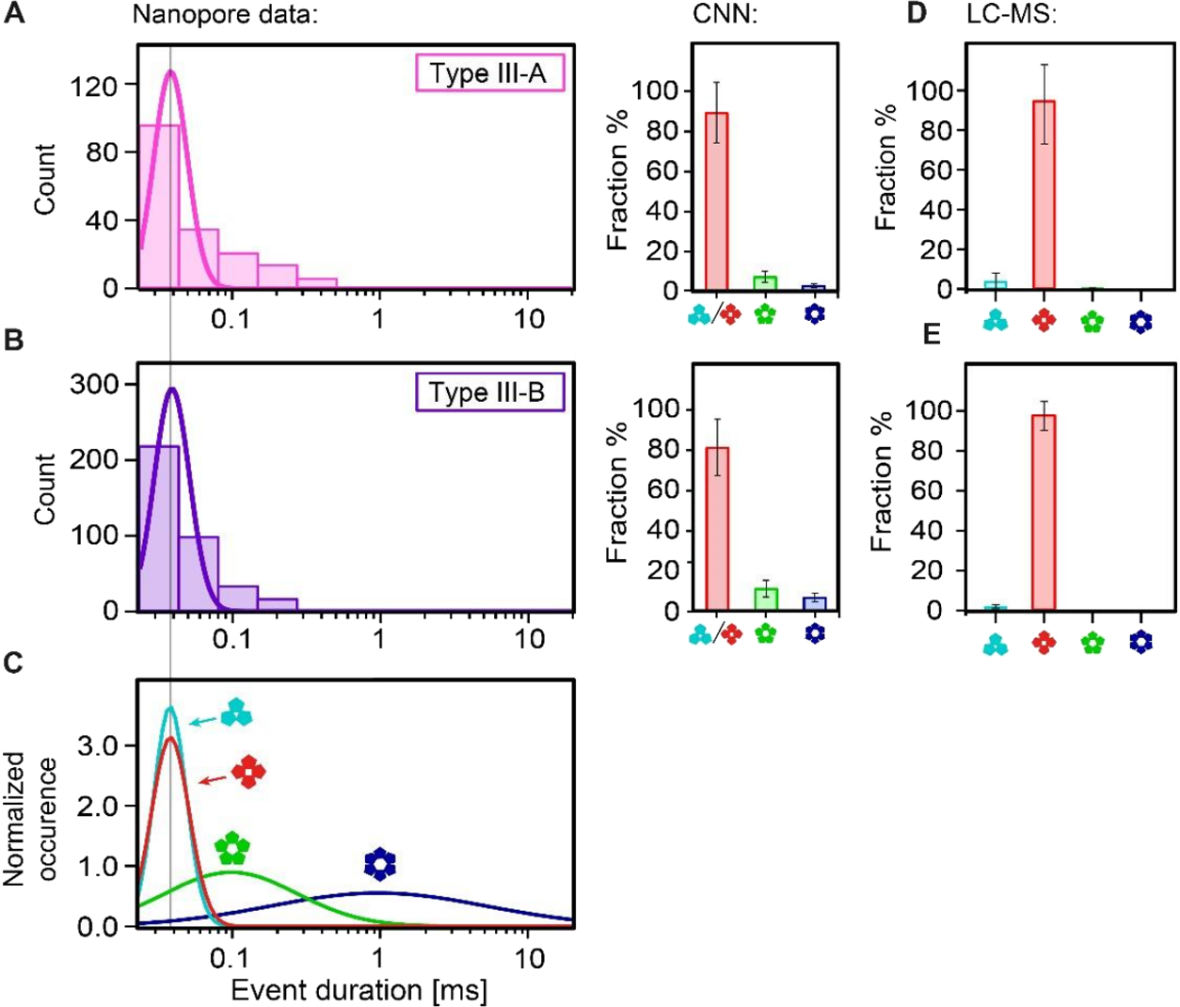
Identification of the cOA composition produced by CRISPR-Cas type III-A and III-B. (A) Histograms of the nanopore event durations measured for the cOAs produced by the type III-A variant (measurement conditions as in Figure 2A–D). The CNN-based quantification of the stoichiometric composition is shown on the right. Error bars denote the identification uncertainty of each cOA class, estimated as 1—the diagonal of the confusion matrix in Figure 3B. (B) Same as (A) but for the cOAs produced by the type III-B variant. (A, B) The nanopore experiments were performed in triplicate. See Figure S6 for the IV curves. (C) Calibration data obtained with monodisperse cOA (cf. Figure 2E). A vertical line through (A–C) provides a guide to the eye. (D, E) LC-MS results type III-A (D) and III-B samples (E), respectively (see the Methods Section). The uncertainties were estimated by error propagation (see the Methods section).

We next turned to biological cOAs, produced *in vitro* by two different CRISPR-Cas variants—type III-A and III-B—that coexist in *T. thermophilus* HB8 (see the Methods Section). If the benefit of having not one but two different type III subtypes in one organism is to activate different CARF proteins, then their cOA composition should differ. We directly tested this hypothesis with nanopore experiments using cOAs produced *in vitro* by reconstituted type III complexes.^15,41^ First, we checked if other substances present in the enzymatic reaction mix, such as ATP or short RNAs (*i.e*., the guide RNA and the target RNA), would interfere with the experiment. However, as they do not produce detectable nanopore signals (Figure S12), they do not interfere with the cOA quantification. Likely, the cyclized structure of the cOAs is essential for their detection, whereas the short noncyclic RNA molecules cannot be resolved because they translocate faster through the nanopore—too fast, in fact, for the time resolution of our experiment (100 kHz, cf. Figure S11). Interestingly, we find that the two CRISPR-Cas subtypes produce nearly identical cOA distributions within the experimental uncertainties (Figure 4A,B). The comparison with the monodisperse calibration data (Figure 4C) shows that cA_3/4_ are the predominant stoichiometries in both cases. Using the pretrained CNN workflow, we quantified the relative abundance of cA_3/4_ as 89 ± 16, and 81 ± 14% for type III-A and III-B, respectively (inferred fraction ± identification uncertainty, see last section). Only minor amounts of cA_5_ (8 ± 3, and 12 ± 5%), and cA_6_ (3 ± 1, and 7 ± 3%) were detected for type III-A and III-B, respectively, which lie at the resolution limit of our technique. In absolute numbers, this converts to 870 ± 420 cOA molecules produced per type III-A complex, and 2800 ± 840 cOAs per type III-B complex under the conditions used (see the Methods Section). We confirmed these results by LC-MS: after validation with known monodisperse and mixture samples (Figures S13–S17), we measured the enzymatically produced cOA from CRISPR-Cas type III-A and III-B (Figures 4D,E, S18, and S19) and found excellent agreement of the results from LC-MS and nanopore detection. Both results consistently show that type III-A and III-B variants both produce nearly identical cOA stoichiometries (within the experimental uncertainties), consisting mainly of cA_4_ and only trace amounts of other cOA as revealed by LC-MS. cA_6_ was indetectable by LC-MS, which lacks single-molecule resolution. In summary, the presented label-free nanopore-CNN workflow revealed that the cOA produced by the Cas10 subunit of *T. thermophilus* type III-A and III-B complexes are very similar (80–90% cA_3/4_), as verified by LC-MS which further revealed cA_4_ as the one predominant species produced by both variants.

### cOAs Produced by CRISPR-Cas Type III-A and III-B Activate a cA_4_-Specific CARF

**Figure 5:**
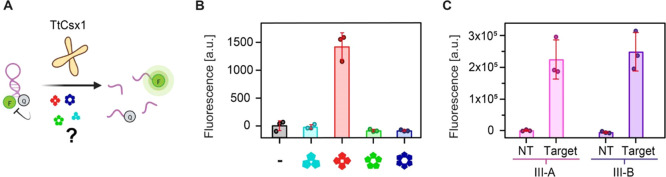
CARF activation by enzymatic and synthetic cOAs reveals stoichiometric specificity. (A) Schematic representation of the RNA cleavage assay. Reporter RNA molecules conjugated with a fluorophore (F) and a quencher (Q) are incubated with the CARF RNase TtCsx1 and various cOA samples. If the cOAs activate TtCsx1, it cleaves the reporter RNA, leading to increased fluorescence intensities compared to the negative controls. (B) Activity assay of TtCsx1 after the addition of synthetic monodisperse cOAs, or no cOAs in case of the negative control (−). Also, cA_2_ was tested and did not activate TtCsx1 (Figure S20). (C) TtCsx1 RNase activity upon addition of cOAs produced in an *in vitro* reaction mixture by endogenous type III-A and III-B from *T. thermophilus*, as indicated. cOAs are only produced in the presence of complementary target RNA (Target). Reactions with noncomplementary target RNA (no target, NT) serve as a negative control for the assay, run in triplicates. (B, C) Error bars denote the standard deviation of three replicates. The raw data (time-dependent fluorescence recordings) are provided in Figure S21. Different spectrometers were used for (B) *vs* (C) yielding different a.u. values. See the Methods Section for details.

After establishing that the type III-A and III-B complexes of *T. thermophilus* produce similar cOAs, we moved on to test their capacity for downstream activation of the CARF protein Csx1 from the same host (TtCsx1). Since this CARF protein has nonspecific RNase activity,^41^ a fluorescent RNA cleavage reporter system was used to screen the activation of CARF proteins by cOAs from synthetic and enzymatic origin (Figure 5A, Methods Section). As expected,^42^ among the synthetic cA_3_ to cA_6_, only cA_4_ led to TtCsx1 activation, resulting in RNA cleavage (Figure 5B). For the cOAs produced by the type III-A and III-B variants (Figure 5C), we found that both activate the RNase activity of TtCsx1 much beyond the negative controls (no target, NT). Hence, Figure 5C further confirms from a functional perspective that both variants (type III-A and III-B) produce cA_4_ capable of CARF activation. Together with the nanopore-CNN and LC-MS data above, our results show that both type III variants produce a nearly identical cOA composition (within the experimental uncertainties), predominantly cA_4_. These results argue against the hypothesis that individual Cas10 homologues produce distinct cOA compositions for a homologue-specific downstream regulation in *T. Thermophilus*. The complementary nanopore-CNN workflow, LC-MS, and activity assays have thus elucidated the hitherto unknown stoichiometric composition of the cOA second messengers of these type III-A and III-B systems, and given the results, the purpose of multiple CRISPR-Cas homologues in one host remains an open question.

## Conclusions

**Figure 6:**
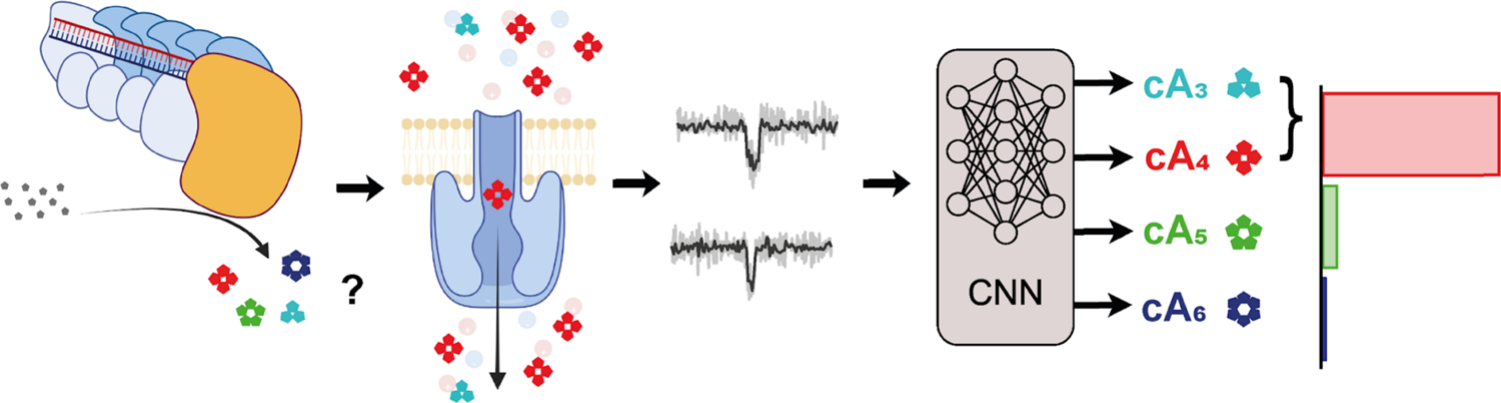
Nanopore-CNN pipeline for the identification of single second messengers from type III CRISPR-Cas. Biological cOAs of unknown stoichiometry, produced by CRISPR type III-A and III-B complexes, are detected one by one in nanopore experiments. Single-molecule events extracted from the nanopore reads are then identified by a pretrained CNN, yielding the stoichiometric composition of the biological cOA samples.

We present a cOA identification workflow with single-molecule resolution (Figure 6) that combines the potential of nanopore technology and neural networks. In this way, we were able to individually detect these small cOA second messenger molecules from the type III CRISPR-Cas systems. Based on the observed nanopore event durations, we could distinguish cA_3/4_ from cA_5_, and cA_6_. Using monodisperse synthetic cOAs, we acquired calibration data for each stoichiometry and used it to train a CNN, which we validated using cOA samples of known polydisperse composition. We then detected cOAs of enzymatic origin, produced by the type III-A and type III-B CRISPR-Cas complexes of *T. thermophilus*, and quantified the previously unknown stoichiometric composition using the validated CNN. These results were further verified by LC-MS. Additional enzyme activity assays revealed the stoichiometry-specific activation of CARF proteins, and further complement the nanopore-CNN results.

We conclude from the nanopore experiments that (i) cyclic oligoadenylates (cA_3_ to cA_6_) can be sensitively detected at the single-molecule level; (ii) the cA_3/4_, cA_5_, cA_6_ stoichiometries cause distinguishable nanopore event durations; and (iii) the type III-A and type III-B CRISPR-Cas complexes produce cOAs of qualitatively near-identical stoichiometry: predominantly the small cA_3_ or cA_4_. Using a pretrained CNN, we (iv) identify the stoichiometries of the cOA at the single-event level with an accuracy of 73%, and we (v) quantify the cOA composition produced by *T. thermophilus* CRISPR-Cas type III-A and type III-B variants as 89 ± 16 and 81 ± 14% cA_3/4_, respectively, and only trace amounts of cA_5_ and cA_6_ in both cases. We also estimate (vi) the absolute number of cOAs produced which amounts to 870 ± 420 cOAs per type III-A complex, and 2800 ± 840 cOAs per type III-B complex, under the conditions used. Lastly, (vii) the enzymatic RNA cleavage assays verified that the combined cA_3,4_ class must contain cA_4_, proving that CRISPR-Cas type III-A and III-B both produce this specific second messenger. This finding was also confirmed by LC-MS identifying over 80% cA_4_ and only traces of other stoichiometries, which is perfectly in line with the nanopore-CNN results. Altogether, we thus find nearly identical stoichiometric cOA compositions in both variants (within experimental uncertainties), which challenges the hypothesis that co-occurring type III complexes would produce distinct cOA for specific downstream regulation purposes.

In the future, the presented nanopore-CNN pipeline can be used to elucidate the cOA stoichiometries of different CRISPR-Cas type III, and it is readily adaptable to additional signaling molecules, which include—but are not limited to—signaling molecules of other antiviral immune systems, such as cyclic oligonucleotide-based antiphage signaling system (CBASS).^43^ Detecting *in vivo* produced cOA from cell lysates is an exciting next step to pursue. Additional protein nanopores will be explored to detect also cA_2_ and to distinguish cA_3_ from cA_4_, which was not possible with our current hemolysin nanopore. Promising candidates include (possibly engineered) pore proteins with a long narrow channel (hemolysin, aerolysin^44^), or with multiple constrictions (CsgG^45,46^). Altogether, we report a single-molecule sensor for CRISPR second messengers with stoichiometry sensitivity. We achieved this using affordable nanopore technology with single-molecule resolution, overcoming the dynamic range limitations of established ensemble techniques. In addition, the presented workflow can be integrated into handheld devices and may, in the future, enable quantitative and accessible point-of-care diagnostics.^47^

## Methods

### Protein Nanopore Recordings

Were performed using the Montal-Mueller technique,^48^ using an Axopatch 200B amplifier and a Digidata 1550B digitizer (both Molecular Devices) with a sampling rate of 500 kHz, and low-pass filtered at 100 kHz. A free-standing lipid bilayer was formed in a custom-built flowcell with two buffer reservoirs separated by a Teflon film (GoodFellow, Huntingdon, England) with a small electro-sparked aperture (*ca.* 100 μm diameter, obtained with a spark generator, Daedalion, Colorado). The Teflon film was pretreated on each side with 10 μL of a hexadecane solution (10% hexadecane, Acros Organics, Geel, Belgium) in pentane (Alfa Aesar, Massachusetts), and let dry for 5 min. Both reservoirs were filled with 400 μL of measurement buffer each (3 M KCl, 100 mM Tris, 0.1 mM EDTA, pH 8), connected to Ag/AgCl electrodes (silver wire with 0.5 mm diameter, Advent, Oxford, England, chloridized in household bleach), and the flowcell was placed inside a Faraday cage. Bilayers were built by adding 10 μL of a 1,2-diphytanoyl-*sn*-glycero-3-phosphocholine solution (DPhPC, 10 mg/mL, Avanti Polar Lipids, Alabama) in pentane onto each buffer reservoir and pipetting up and down as described previously.^48^ α-HL oligomers (kindly provided by Sergey Kalachikov, Columbia University) were added to the trans reservoir (with the working electrode under positive polarity) and their spontaneous membrane insertion caused a characteristic current. The synthetic cOA solutions (500 μM, Biolog, Bremen, Germany) were added to the cis reservoir (with the ground electrode) to a final concentration of 10 μM. For the cOA produced by CRISPR-Cas type III-A and III-B, the sample volumes added to the cis reservoir were 20 and 15 μL, respectively. All experiments were performed in triplicate, with individual pores. See Figure S6.

### Nanopore Data Processing

was performed in Igor Pro (v6.37, Wavemetrics, Oregon) using custom code. Event detection was performed after filtering the signal with a digital 80 kHz low-pass filter and median-conserving decimation to a final sampling rate of 80 kHz (25 μs time resolution). Events were extracted by applying a threshold at 65% of the open-pore current. The mean current blockades were calculated from the extracted data. To calculate event durations, 1000-fold bootstrapping with replacement was performed, where each subset was fit with a single-exponential (with X offset). The uncertainty is expressed as the standard deviation of all bootstrapped time constants.

### Neural Networks: Per-Event Classification

To classify single cOA translocation events, we trained a one-dimensional (1D) convolutional neural network (CNN) implemented using TensorFlow.^49^ As training data, we used 48 traces of +120 mV translocations from synthetic monodisperse cOA samples with known stoichiometries. Events were extracted as described above, individually normalized, and scaled by the standard deviation. To provide information about the relative blockade, we include 47 data points (5875 ms) of the baseline signal on either side of the extracted event. Moreover, to ensure consistent input size, we pad each to a width of 250 data points with zeroes on the right-hand side. The original event duration is concatenated to this signal as a final feature.

We feed each individual padded event into the CNN, which performs multiclass classification. The CNN consists of two 1D convolutional layers with 10 filters of width 25 and a ReLu activation function, each of which is followed by batch normalization and 20% dropout operations. Next, values are maxpooled with a pool size of 10, and fed into a dense layer with 4 output nodes (one per cOA class) and a softmax activation function. The CNN is trained for 100 epochs at a learning rate of 0.001 with the Adam optimizer that minimizes cross-entropy loss based on the 3 one-hot encoded cOA classes. During training, we perform oversampling to prevent class imbalance. Five restarts were performed, after which the classifier with the highest training accuracy was selected for testing. Training was done on a laptop equipped with an Intel Core i7 processor (8 cores @3 GHz, Santa Clara, CA) and a T500 GPU (NVIDIA, Santa Clara, CA). Training runtime was around 20 min.

### CNN Validation

was done using a 10-fold cross-validation scheme. Importantly, events of a given trace were never split between training and test sets, thus ensuring that no trace-specific characteristics were learned. The reported overall accuracy was calculated over the predictions of all folds merged into one data set. We compared the CNN performance to a traditional feature-extraction-based classifier by performing k-nearest neighbor (KNN) classification based on relative blockade and (log-transformed) dwell times (*k* = 3 after hyperparameter optimization), implemented using sci-kit learn.^50^ In cOA mixture classifications, *t*-tests and two one-sided *t*-tests (TOST) procedures were used to test difference and equivalence respectively between all cOA distributions. The TOST procedure tests whether two cOA relative distributions *N*(μ_cA*x*_,σ_cA*x*_)and *N*(μ_cA*y*_,σ_cA*y*_) have means diverging less than a given level δ, by performing two one-sided *t*-tests, with null-hypotheses
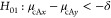

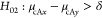
where rejection of both *H*_01_ and *H*_02_ means that the alternative hypothesis that the means differ less than δ must be accepted, or
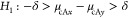


Both methods are implemented in python using the scipy package^51^ and available in the main code repository for this paper (see Code availability).

### Abundance Estimation

ability was evaluated by running inference using the trained model on polydisperse samples of known composition. To obtain prediction intervals reflecting variation induced by training data, prediction was repeated with all 10 classifiers obtained during cross-validation. We compensate for differences in event rate by using cOA-specific constants which convert from event count to estimated abundance in a sample. The constants are estimated by counting the number of events in the training files and normalizing for concentration and time. The values of these correction factors (in units of events s^–1^ μMol^–1^) were 0.17 ± 0.05, 0.62 ± 0.7, and 0.12 ± 0.3 for cA_3/4_, cA_5_, and cA_6_, respectively.

### Enzymatic cOA Synthesis and Quantification

Either TtCsm (type III-A) or TtCmr (type III-B) endogenous protein complexes from *T. thermophilus* (purified as described previously^15^) were incubated in 150 mM NaCl, 20 mM Tris-HCl pH 8, 10 mM DTT, 2 mM MgCl and 1 mM ATP in a total volume of 20 μL, to which 200 nM of nontarget or target RNA (IDT, Coralville, Iowa) was added, as indicated. The reactions were carried out in triplicate by incubating the samples for 60 min at 65 °C. Target RNA sequence: 5′ GAACUGCGCCUUGACGUGGUCGUCCCCGGGCGCCUUAUCUACGGCCAUCG 3′.

Nontarget RNA sequence: 5′ UGAUGAGGUAGUAGGUUGUAUAGUAAGCUUGGCACUGGCCGUCGUUUACG 3′.

The absolute numbers of each cOA produced by the type III-A and III-B complexes were calculated from the observed nanopore event rate *r*_e_ (in units of s^–1^), the CNN deduced fraction cA_*x*_% of each stoichiometry class “*x*”, and the predefined correction factor CF_*x*_ (see last section), the dilution factor *d* (between the enzyme reaction and the nanopore experiment), and the Avogadro constant *N*_A_
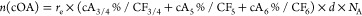


For the type III-A and III-B measurements, the respective event rates were 0.47 ± 0.19 and 1.17 ± 0.11 s^–1^, and the dilution factors were *d* = 21 and *d* = 27.6, respectively. We report the cOA numbers normalized per type III-A or III-B complex (their concentration during cOA synthesis was 62.5 nM), and the uncertainty was calculated by error propagation of the reported individual uncertainty estimates.

### CARF cOA Stringency Assay

The CARF protein Csx1 from *T. thermophilus*, 1 μM TtCsx1, purified as described previously,^41^ was incubated with 250 nM RNaseAlertTM (ThermoFisher, Waltham) in 150 mM NaCl, 20 mM Tris-HCl pH 8, 10 mM DTT, and 1 mM ATP in a total volume of 20 μL, to which 1 μM of synthetic cOAn (*n* = 2–6, Biolog, Bremen, Germany) was added. The reactions were carried out in triplicate for 60 min at 65 °C in a Bio-Rad CFX384TM Real-Time System (Hercules), measuring FAM at 1 min intervals. Data shows the relative fluorescence at 30 min.

### CARF Activation Assay with Enzymatic cOA from Type III CRISPR

Either type III-A or type III-B endogenous complexes from *T. thermophilus* were incubated with 1 μM TtCsx1 (the CARF protein), 250 nM RNaseAlertTM (ThermoFisher, Waltham) in 150 mM NaCl, 20 mM Tris-HCl pH 8, 10 mM DTT, 2 mM MgCl and 1 mM ATP in a total volume of 20 μL, to which 200 nM of nontarget or target RNA (IDT, Coralville, Iowa) was added, as indicated. The reactions were carried out in triplicate for 60 min at 65 °C in a Thermo Fisher QuantStudio 1 Real-Time PCR System (Waltham). Data shows the relative fluorescence at 30 min. One μM of cA4 was used as a positive control in this assay. Target RNA sequence: 5′ GAACUGCGCCUUGACGUGGUCGUCCCCGGGCGCCUUAUCUACGGCCAUCG 3′. Nontarget RNA sequence: 5′ UGAUGAGGUAGUAGGUUGUAUAGUAAGCUUGGCACUGGCCGUCGUUUACG 3′.

### HPLC-MS Measurements

The cOAs were separated, as previously described,^9^ on a Thermo Scientific Dionex UltiMate 3000 (RS) HPLC, equipped with a Kinetex 2.6 μm Polar C18 100A (150 mm × 4.6 mm, Phenomenex) column. Four mM ammonium bicarbonate in water was used as mobile phase A and acetonitrile was used as mobile phase B. A multistep gradient was applied as shown in Table S3 at a constant flow rate of 0.35 mL/min and a column temperature of 40 °C. Moreover, an injection volume of 5 uL was used. High-resolution MS data was acquired on a Bruker maxis 4G ESI QTOF in negative ion mode with a scan range of *m*/*z* 75–1700. The source voltage was set to 4.5 kV, the nebulizer pressure was set to 0.4 bar, and the dry gas temperature was 180 °C (flow 4.0 l/min). The acquired data was processed with Bruker DataAnalysis 4.4. Both monodisperse and polydisperse cOA samples of known composition were used to calibrate and validate the LC-MS setup for cOA detection: The monodisperse cOA solutions (cA_3_ to cA_6_) were prepared at a concentration of 50 μM in the reaction buffer (150 mM NaCl, 20 mM Tris-HCl pH 8, 10 mM DTT, 2 mM MgCl and 1 mM ATP) and measured in duplicate to calibrate LC-MS cOA signals prior to the measurement of the mixtures, the type III-A, and III-B samples (Figures S13–S16). The validation mixtures were prepared by mixing equal volumes of the 50 μM monodisperse cOA solutions, resulting in equimolar cOA levels (12.5 μM each). The mixtures were then measured by LC-MS in triplicate, and the cOA concentrations were estimated (Figure S17). For this, the extracted ion chromatogram (EIC) peak areas of the main species [M – 2H]^2–^ for all four cOAs in the monodisperse solutions were used as estimators of the concentration of cOAs present in the validation mixture as follows:
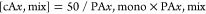
where [cA*x*, mix] is the estimated concentration of each cOA type in the mixture, PA*x*; mono and mix refer, respectively, to the peak areas of the EICs obtained for each cOA in either the monodisperse or the mixture sample LC-MS analysis; and 50 is the concentration (in μM) of each cOA in the monodisperse solutions. The ratios in the mixtures were then calculated as
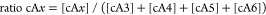
where [cA3], [cA4], [cA5], and [cA6] refer to the concentration of each cOA in the mixture, calculated as indicated above. Uncertainty estimates were obtained by error propagation.

## Data Availability

CNN training and evaluation code is freely available on Gitlab at https://github.com/cvdelannoy/coa_classifier.

## Supporting Information Available

Concentration and voltage effect on cOA capture (Figures S1 and S2); comparison between α-HL and MspA nanopores and performance of MspA for cOA detection (Figures S3 and S4); pore orientation effect on cOA events (Figure S5); performance of the α-HL pores used within this study (Figure S6); representation of a partially folded cOA (Figure S7); aligned nanopore translocation events and event scatterplots (Figures S8 and S9); kNN *vs* CNN classifier comparison (Figure S10); event duration distributions of classified events (Figure S11); negative control for ATP and RNA nanopore signals (Figure S12); LC-MS analysis validation with monodisperse and polydisperse synthetic cOA (Figures S13–S17); LC-MS analysis of biological cOA samples (Figures S18 and S19); cA_2_ activity assay (Figure S20); CARF activity assays time-dependent fluorescence data (Figure S21); cOA event durations at different voltages (Table S1); statistical tests for CNN classification of cOA mixtures (Table S2); and multistep gradient used during LC-MS measurements (Table S3) (PDF)

## Notes

A preprint version of this article is available on bioRxiv: Fuentenebro Navas, D.; Steens J. A.; de Lannoy, C.; Noordijk, B.; de Ridder, D.; Staals, R.H.J.; Schmid, S. Nanopores Reveal the Stoichiometry of Single Oligo-Adenylates Produced by Type III CRISPR-Cas, **2023**. 2023.08.18.553839, *bioRxiv*. 10.1101/2023.08.18.553839 (accessed on May 27, 2024).
