# Supplementary material for: Nanopores Reveal the Stoichiometry of Single Oligoadenylates Produced by Type III CRISPR-Cas
Source: ACS Nano. 2024 Jun 14;18(26):16505–15. doi: 10.1021/acsnano.3c11769 (PMC11223493; doi:10.1021/acsnano.3c11769)
Supplement: Supplementary file 1 — nn3c11769_si_001.pdf [file nn3c11769_si_001.pdf]

Supporting information:

Nanopores reveal the stoichiometry of single oligo-adenylates produced by type III CRISPR-Cas

David Fuentenebro Navas[1], Jurre A. Steens[2], Carlos de Lannoy[3,4], Ben Noordijk[3], Michael Pfeffer[5], Dick de Ridder[3], Raymond H.J. Staals[2], Sonja Schmid[1]*

1) Laboratory of Biophysics, Wageningen University and Research, Stippeneng 4, 6708WE Wageningen, The Netherlands
Present address: Department of Chemistry, University of Basel, Mattenstrasse 22, 4058 Basel, Switzerland

2) Laboratory of Microbiology, Wageningen University and Research, Stippeneng 4, 6708WE Wageningen, The Netherlands

3) Bioinformatics Group, Wageningen University and Research, Droevendaalsesteeg 1, 6708PB Wageningen, The Netherlands

4) Department of Bionanoscience, Delft University of Technology, Van der Maasweg 9, 2629HZ Delft, The Netherlands

5) Department of Chemistry, University of Basel, Mattenstrasse 22, 4058 Basel, Switzerland

*Correspondence: sonja.schmid@unibas.ch

Table of contents:

Figure S1: The concentration-dependent cOA event rates (3).

Figure S2: The voltage-dependent event rates and durations (3).

Figure S3: The MspA nanopore detects cOAs with shorter event durations than α-HL (4).

Figure S4: Performance of the MspA nanopore for cOA detection at 3M KCl (5).

Figure S5: Comparison of the cis vs trans-inserted α-HL nanopore regarding its cOA detection capacity (6).

Figure S6: IV curves (current vs voltage) of the α-HL pores used in this study. (7).

Figure S7: cOAs have a variable diameter in solution (8).

Figure S8: Aligned nanopore translocation events of cOA (9).

Figure S9: 2D scatter plot of the nanopore translocation events of the indicated cOA molecules (10).

Figure S10: k-nearest neighbor performance on cOA event classification (11).

Figure S11: Event duration distributions of unseen nanopore events, per ground truth and CNN-predicted stoichiometry (12).

Figure S12: Negative control: neither ATP nor RNA from enzymatic reactions cause detectable nanopore events. (13).

Figure S13: cA_3 LC-MS data (14).

Figure S14: cA_4 LC-MS data (15).

Figure S15: cA_5 LC-MS data (16).

Figure S16: cA_6 LC-MS data (17).

Figure S17: Validation of the cOA analysis by LC-MS (18).

Figure S18: LC-MS results of the cOA produced enzymatically by CRISPR-Cas type III-A (19).

Figure S19: LC-MS results of the cOA produced enzymatically by CRISPR-Cas type III-B (20).

Figure S20: cA_2 does not activate TtCsx1 (21).

Figure S21: Time-dependent fluorescence data of the CARF activity assays (22).

Table S1: Nanopore event durations of cA_{3-6} at different voltages (23).

Table S2: Pair-wise statistical difference and equivalence test p-values, for neural network classifications of cOA mixtures (24).

Table S3: Multi-step gradient used during LC-MS measurements (25).

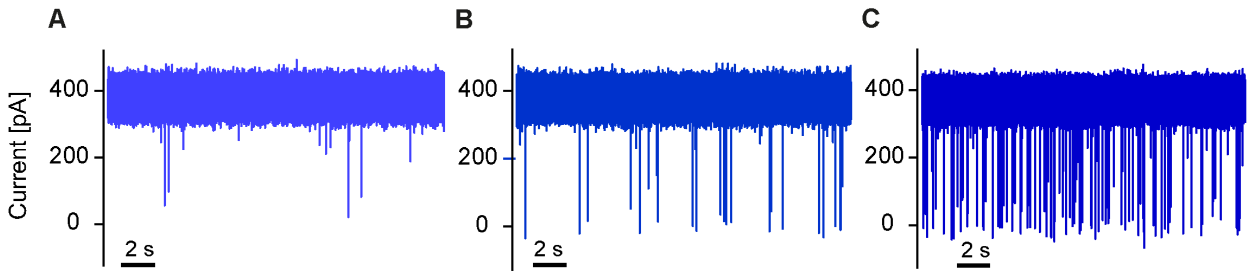

Figure S1: Concentration-dependent cOA event rates. Current traces recorded with a trans-inserted α-HL pore at +160mV in a 3M KCl buffer (Methods) after the addition of cA_6 to the cis chamber: **A)** 100nM, **B)** 1μM, **C)** 10μM cA_6. The event rates are: 0.2, 0.7, and 3.8 events/s, respectively. cA_6 events are also detectable below 100nM, albeit at correspondingly slower event rates.

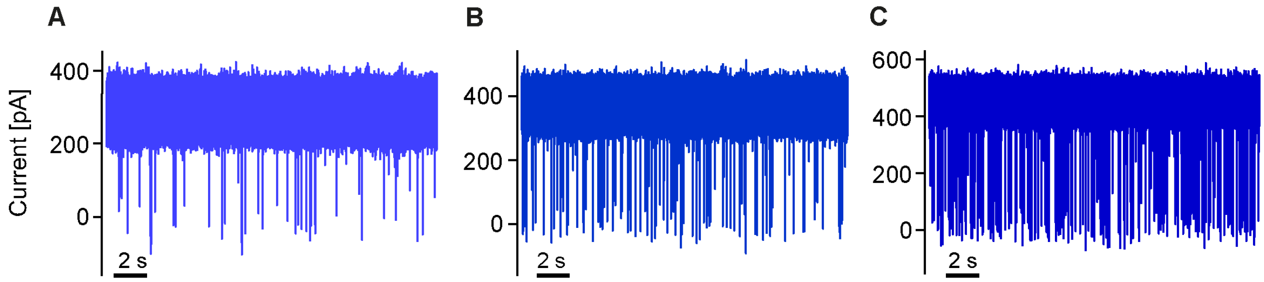

Figure S2: Voltage-dependent event rates and durations. Current traces recorded with a trans-inserted α-HL pore in a 3M KCl buffer (Methods) after the addition of 10μM cA_6 to the cis chamber, recorded at **A)** +120mV, **B)** +160mV, **C)** +200mV. As expected for electrophoresis-driven translocation events, the event rate increases with voltage (1.7, 4.3, and 7 events/s, respectively) while the event duration decreases. The voltage-dependent event durations for cA_3, cA_4, cA_5, cA_6 are specified in Table S1.

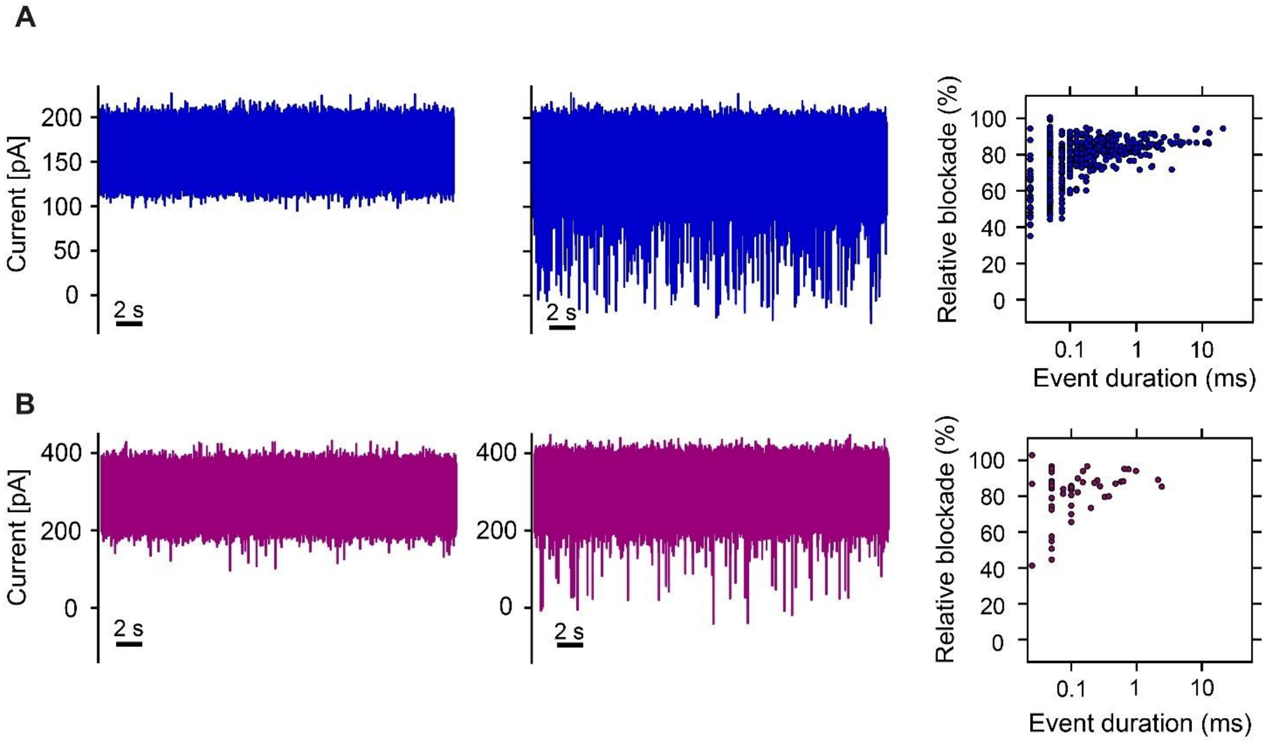

Figure S3: The MspA nanopore detects cOAs with shorter event durations than α-HL. **A)** α-HL. **B)** MspA. Both A, B) were compared under identical conditions at 1M KCl (Methods). Left: baseline current at +160mV of a cis-inserted pore, before analyte addition. Middle: current trace recorded at +160mV after the addition of 10μM cA_6 to the cis chamber. Right: Scatter plot of relative blockade vs. event duration. MspA detects fewer events that are often truncated, and overall shorter than those with α-HL, making the latter a more suitable choice for cOA detection. MspA recordings measured at 3M KCl are shown in Figure S4.

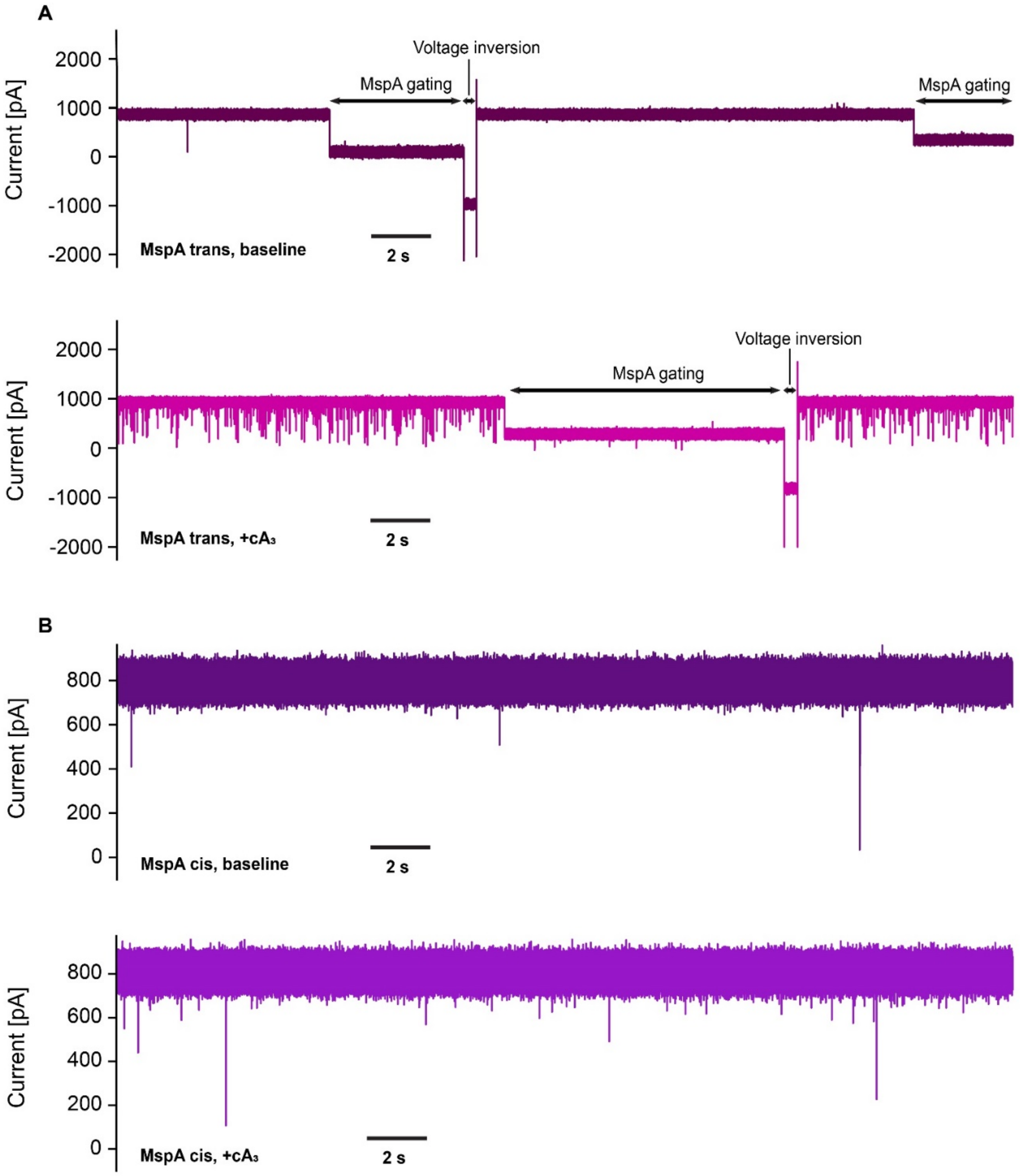

Figure S4: Performance of MspA nanopore for cOA detection at 3M KCl. A) Current recordings of a trans-inserted MspA pore show frequent gating and yield truncated cA_3 events (same conditions as Figure 2 main text: 3M KCl, +120mV). Top: baseline trace, no analyte. Bottom: after addition of 10 µM cA_3 to the cis compartment. Frequent voltage inversion is needed to recover the baseline current after gating. **B)** Current recordings of a cis-inserted MspA pore do not resolve cA_3 (same conditions as Figure 2 main text: 3M KCl, +120mV). Top: baseline trace, no analyte. Bottom: after addition of 10 µM cA_3 to the cis compartment. MspA cannot detect cA_3 in this orientation. These data show that MspA is less suitable for cOA detection than α-HL.

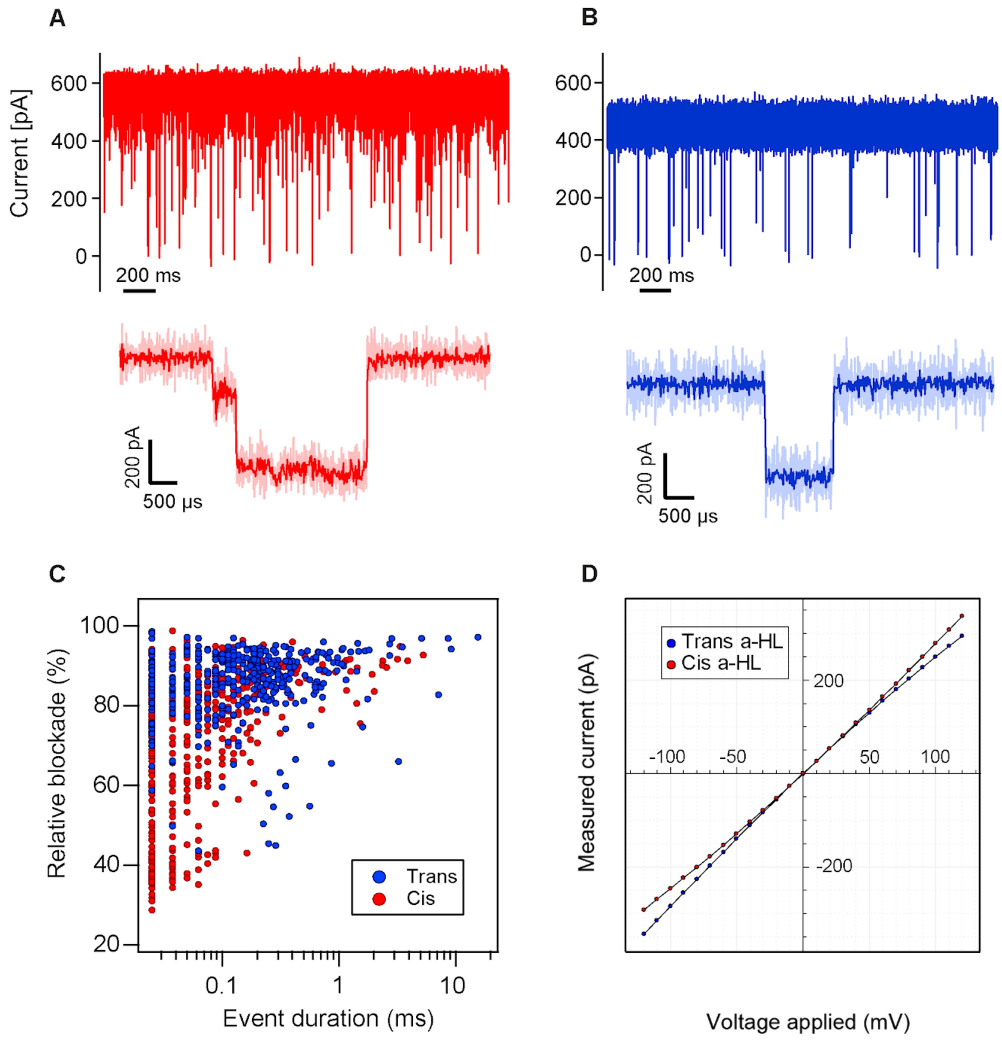

Figure S5: Comparison of the cOA detection capacity of cis vs. trans-inserted α-HL nanopores. A,B) show a current trace and event zoom-in recorded with a cis- (A) or trans-inserted α-HL pore (B), respectively, both measured at +200mV, in 3M KCl, with 10µM cA_6 in cis. The events observed with a cis-inserted α-HL were less uniform. **C)** Scatter plot of blockade vs. duration for the events cis in A and trans events in B, respectively. The events of the cis-inserted α-HL (red) are more heterogenous (spread out) than the more uniform ones of the trans-inserted pore (blue). **D)** Current-voltage curves of cis- and a trans-inserted α-HL pores as indicated (without cOA).

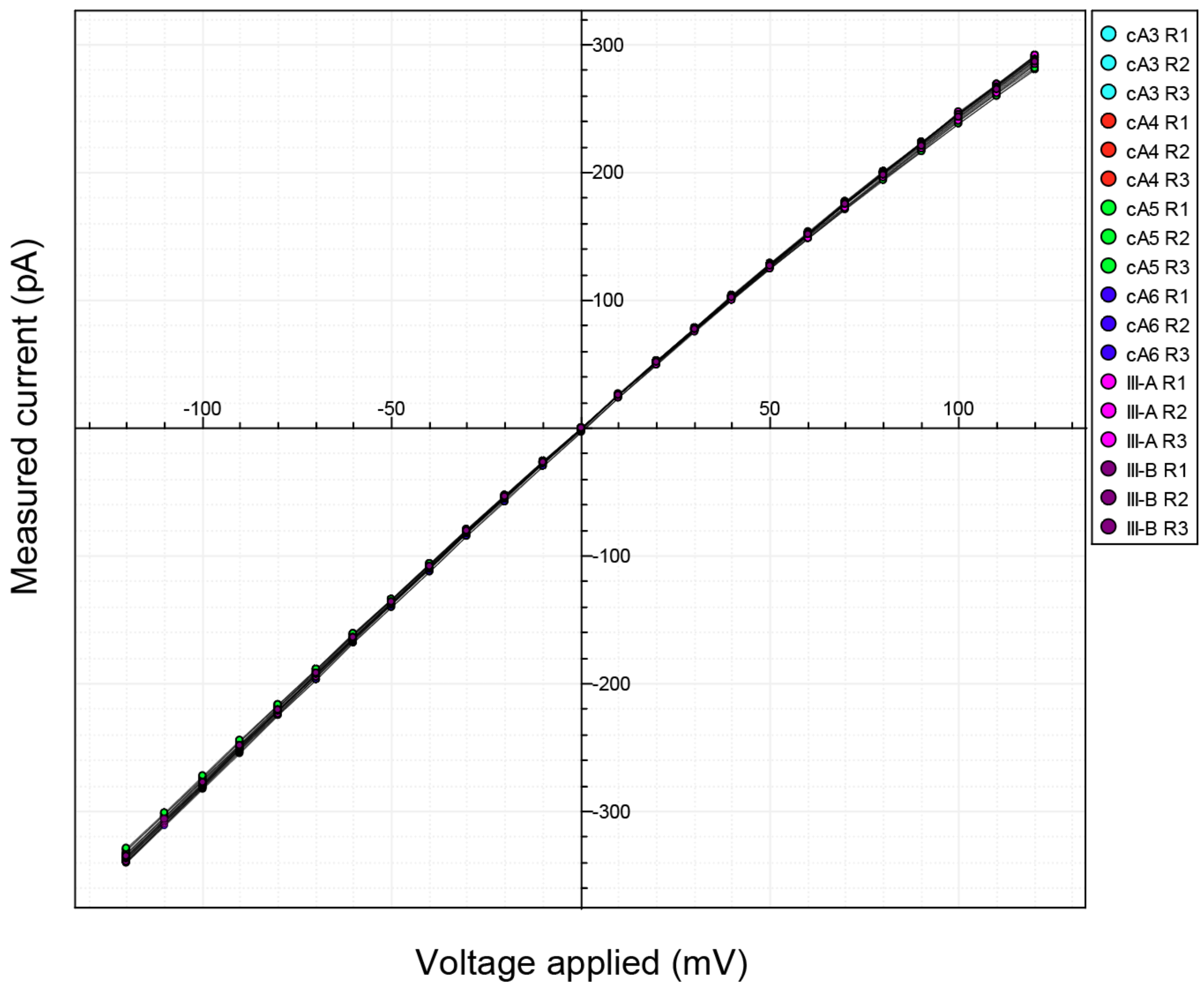

Fig S6: IV curves (current vs voltage) of the α-HL pores used in this study. The IV curves were measured from -120mV to +120mV in 3M KCl before the start of each experiment. They are consistent for all experiments (see legend) and measurement days, and show the typical slight rectification of α-HL.

A

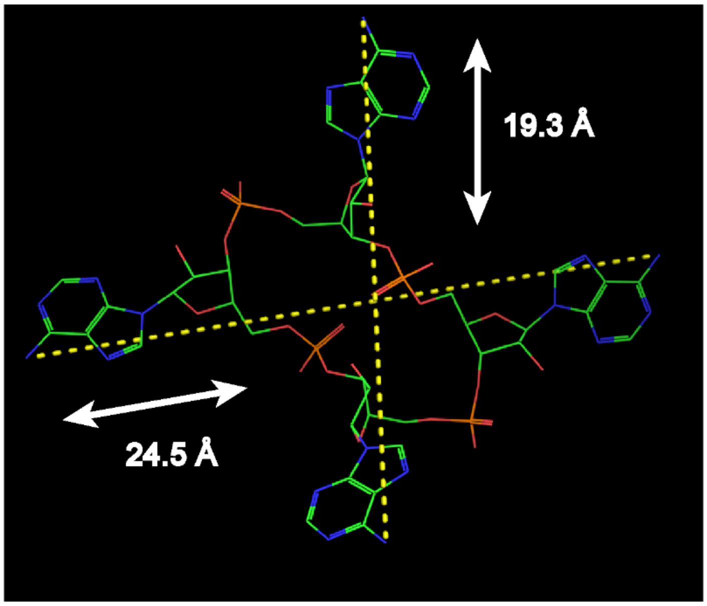

B

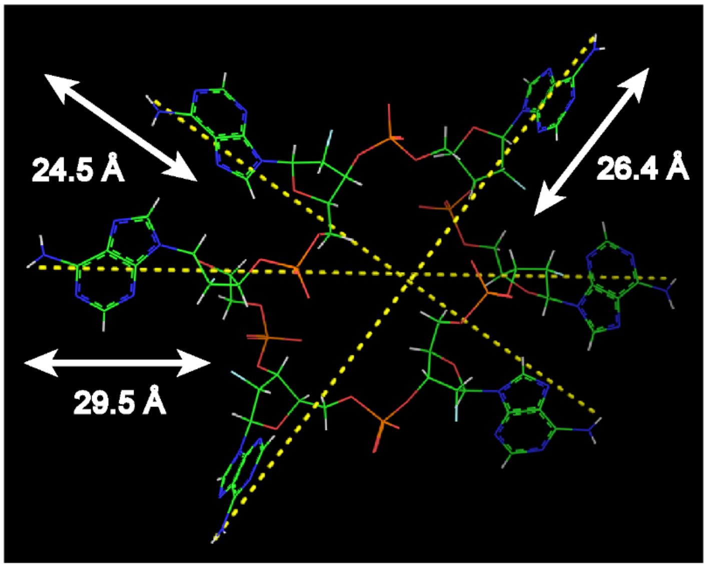

Figure S7: cOAs have a variable diameter in solution. 3D structures of cA_4 and cA_6 in complex with **A)** SsCsa3 (PDB ID: 6W11) and **B)** EiCsm6 (PDB ID: 6TUG) proteins, respectively. The proteins have been hidden for visualization purposes. The cOA have flexible bonds and fold in solution. This leads to a variable diameter, which allows them to squeeze through the narrower α-HL pore (1.2 nm). Note that the molecule shown at the bottom is cyclic hexa-2'-fluoro-hexa-dAMP (cFA_6), a cA_6 mimic resistant to degradation but still able to activate its substrate EiCsm6.

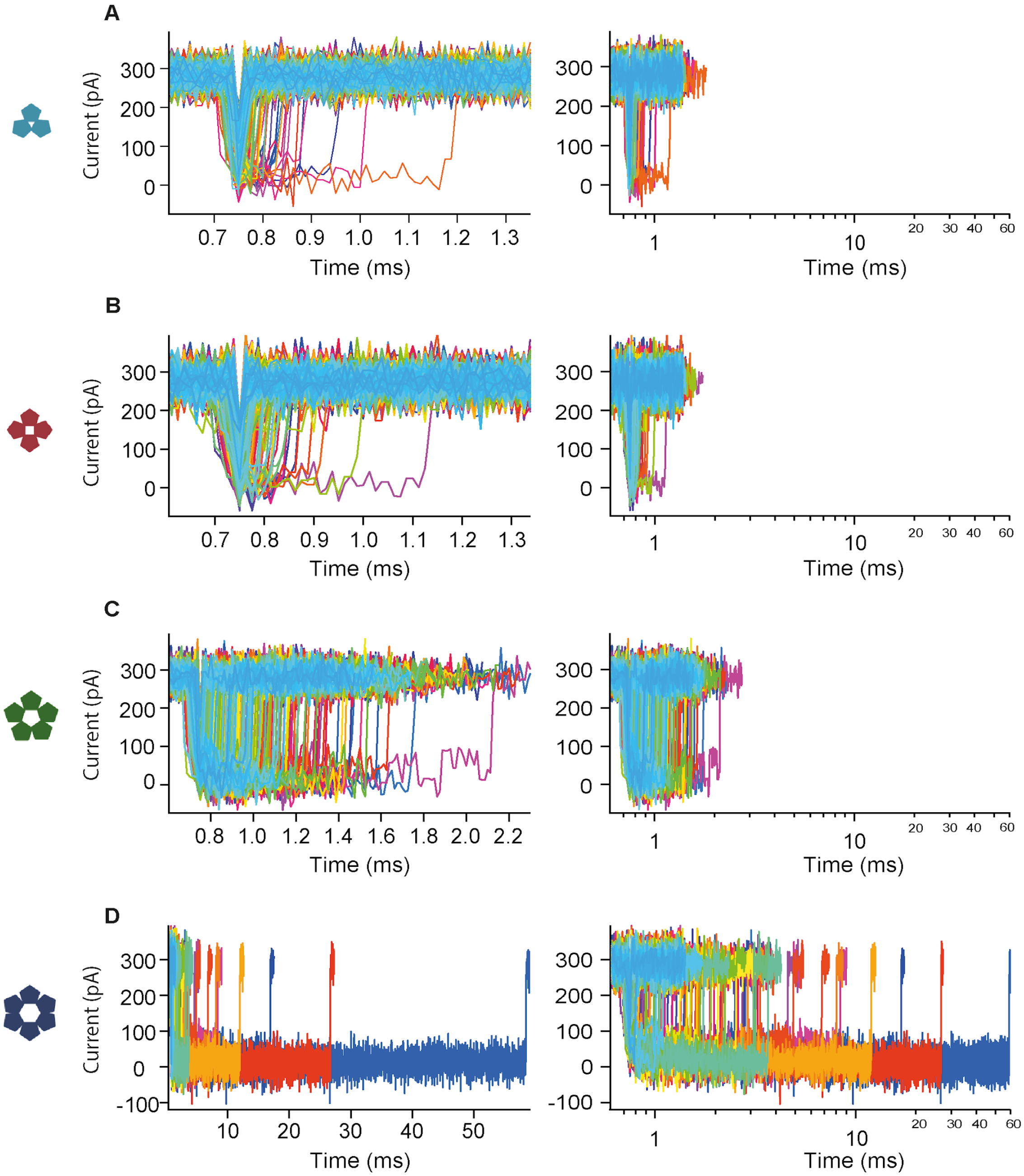

Figure S8: Aligned nanopore translocation events of cOAs: A) cA$_3$, B) cA$_4$, C) cA$_5$, D) cA$_6$. The events were obtained as in Figure 2A (10μM cOA in cis chamber, trans-inserted α-HL pore, +120mV, 3M KCl buffer). The events are aligned at their starting point and individually colored. Plots with linear (right) and logarithmic (left) time axes are shown.

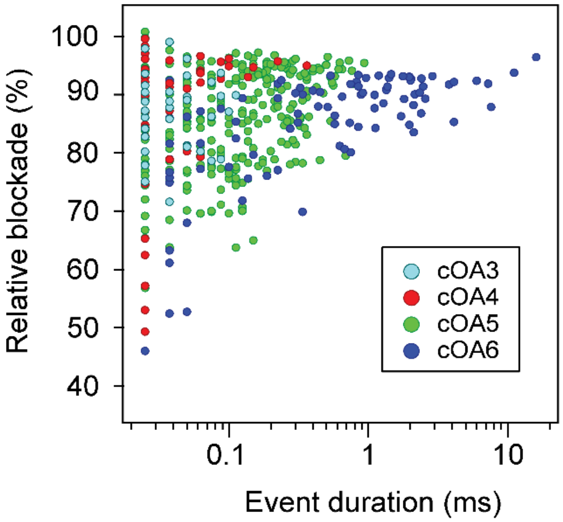

Figure S9: 2D scatter plot of the nanopore translocation events of the indicated cOA molecules. Each dot represents a single translocation event, represented by its relative current blockade vs. its duration.

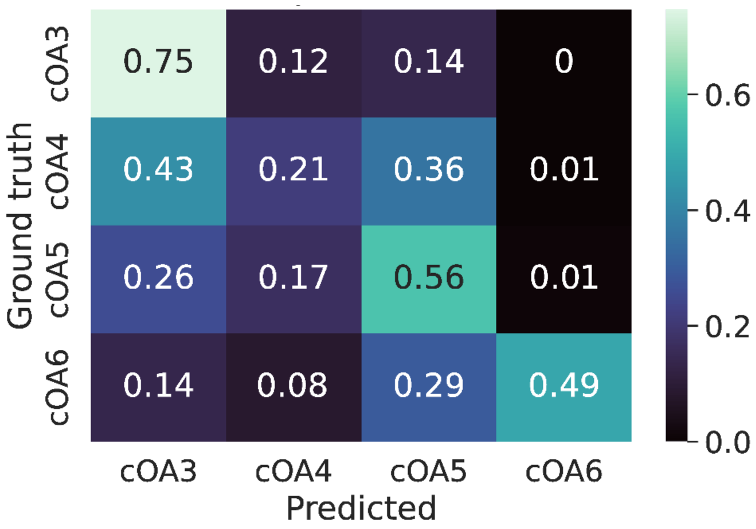

Figure S10: k-nearest neighbor performance on cOA event classification. This confusion matrix shows the performance of a k-nearest neighbor (kNN) classifier for cOA event identification, fed with the same data as the CNNs (Fig 3B). The kNN only uses current blockade and event duration as features for classification, and performs poorly when compared to the trained CNN. This is likely due to the fact that the CNN is able to recognize more subtle patterns present in the signal, such as event shape and current fluctuations, leading to a better classification of unseen events.

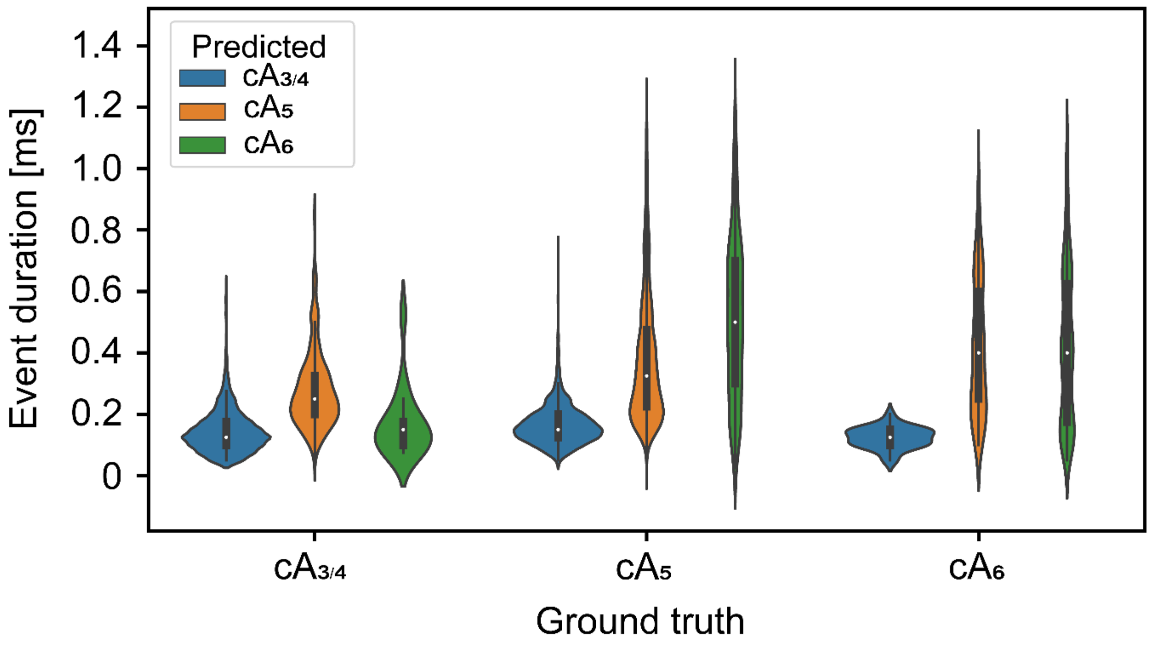

Figure S11: Event duration distributions of unseen nanopore events, per ground truth and CNN-predicted stoichiometry. Test events from mono-disperse samples were classified using the trained CNN and subsequently analyzed on event duration for each combination of ground truth and CNN-predicted stoichiometry. Distributions are shown as violin plots, where each violin surface is normalized to the same surface area to allow for visual distribution comparison. Events that were erroneously predicted to be of lower-stoichiometry than indicated by the ground truth showed shorter event durations, indicating that this feature played a major role in misclassifications.

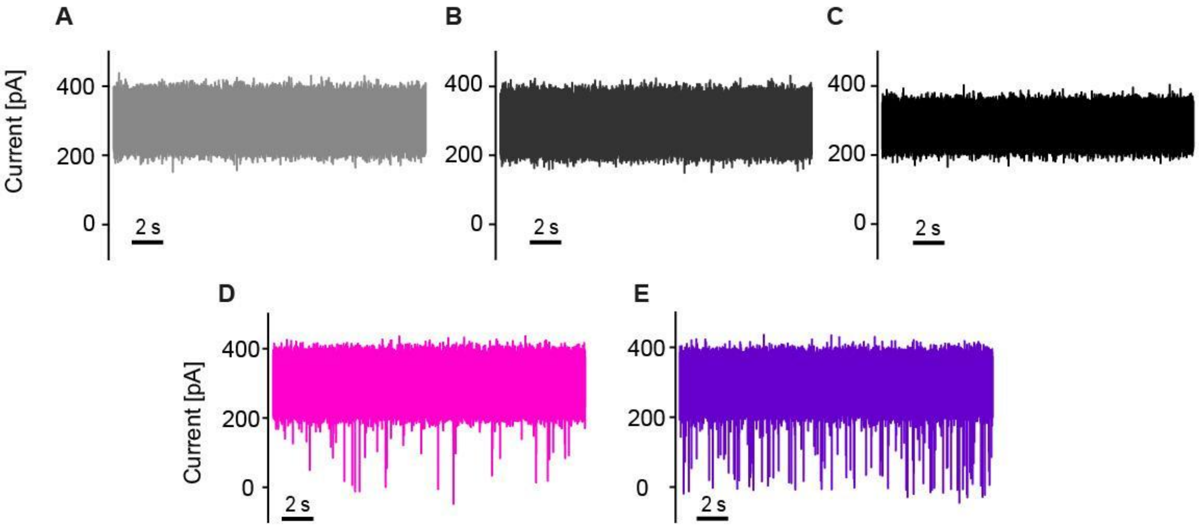

Figure S12: Negative control: neither ATP nor RNA from enzymatic reactions cause detectable nanopore events. All current traces were recorded with a trans-inserted α-HL pore at +120mV in 3M KCl buffer. **A)** Baseline without analyte. **B)** 1µM ATP in the cis chamber (as used in Fig. 4B,C, cf. Methods) does not cause any detectable events. **C)** The enzyme reaction mix (incl. enzyme, RNA, as used in Fig. 4B,C, see Methods) does not cause any detectable events in the *absence* of ATP (precluding enzymatic cOA synthesis). B) and C) confirm that the events detected in Figure 4B,C originate from bona-fide Cas10-produced cOAs, and not from other molecules present in the reaction. **D, E)** Current traces recorded with the *active* reaction mix (incl. ATP) show typical cOA events, indicating the presence of enzymatically produced cOA, synthesized by CRISPR/Cas type III-A (panel D) and III-B proteins (panel E), respectively.

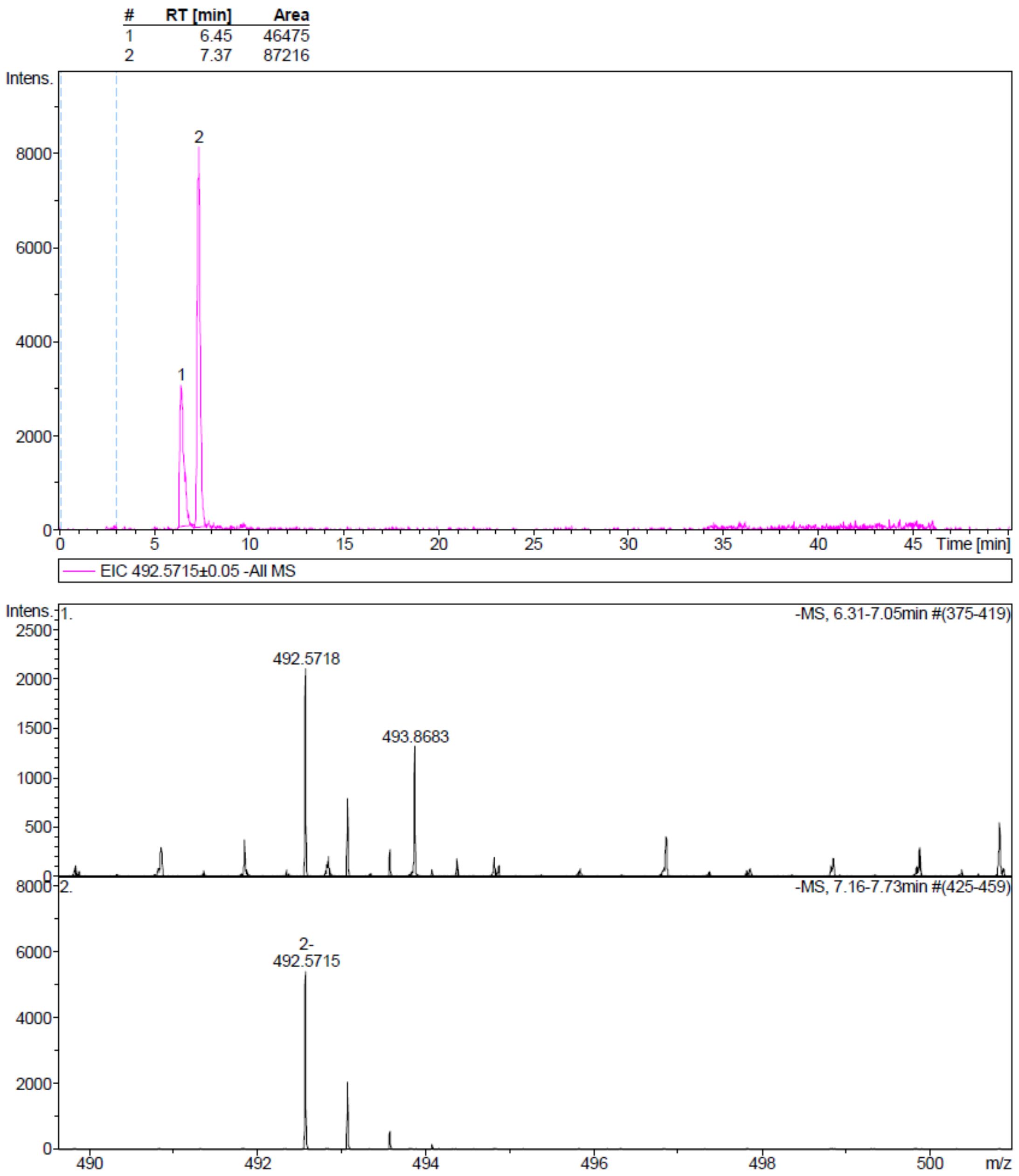

Figure S13: cA_3 LC-MS data. Extracted Ion Chromatograms (EIC) obtained by LC-MS for 50µM monodisperse cA_3 for calibration (Methods). The main species represented for cA_3 is the [M-2H]2-, with a theoretical m/z value of 492.5715, which is in good agreement with the values measured experimentally. The trimer elutes mainly at 7.37 min, with a lower but significant elution at 6.45 min. Both peaks were taken into account for the calibration, as they contained the trimer. The blue dashed line shows the latest elution time for most of the salts and buffer components present in the sample. The calibration was measured in duplicate, right before the the validation mixtures, the type III-A and type III-B samples.

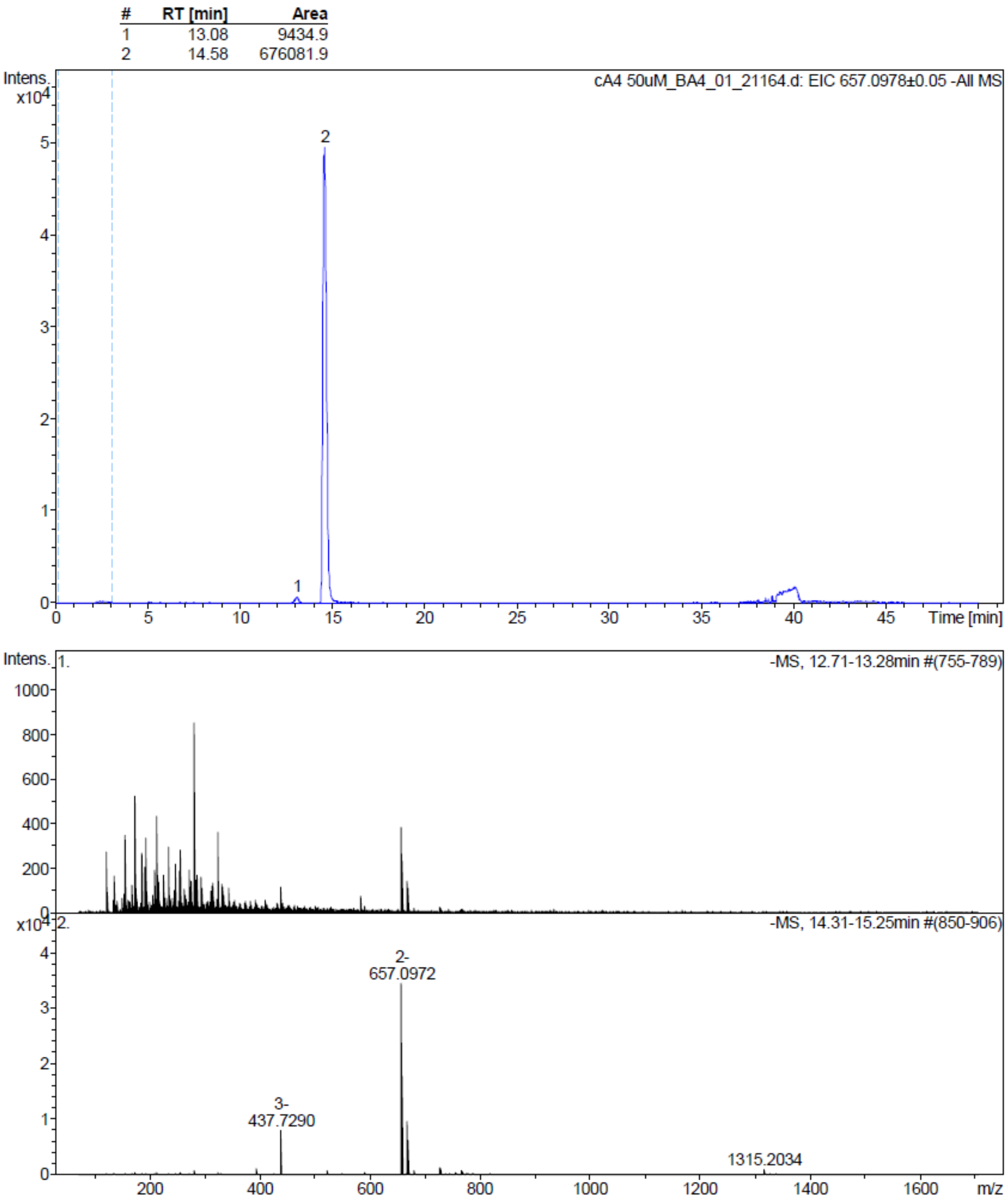

Figure S14: cA4 LC-MS data. Extracted Ion Chromatograms (EIC) obtained by LC-MS for 50µM monodisperse cA_4 for calibration (Methods). The main species represented for cA_4 is the [M-2H]2-, with a theoretical m/z value of 657.0978, in good agreement with the experimental values. The [M-3H]3- (m/z 437.7290) and [M-1H]1- (m/z of 1315.2034) species are also observed, although in lower proportion. The trimer elutes mainly at 14.58 min, with trace amounts eluting at 13.08. The two peaks were taken into account for the calibration, as the tetramer was detected in both. The blue dashed line shows the latest elution time for most of the salts and buffer components present in the sample. The calibration was measured in duplicate, right before the validation mixtures, the type III-A and type III-B samples.

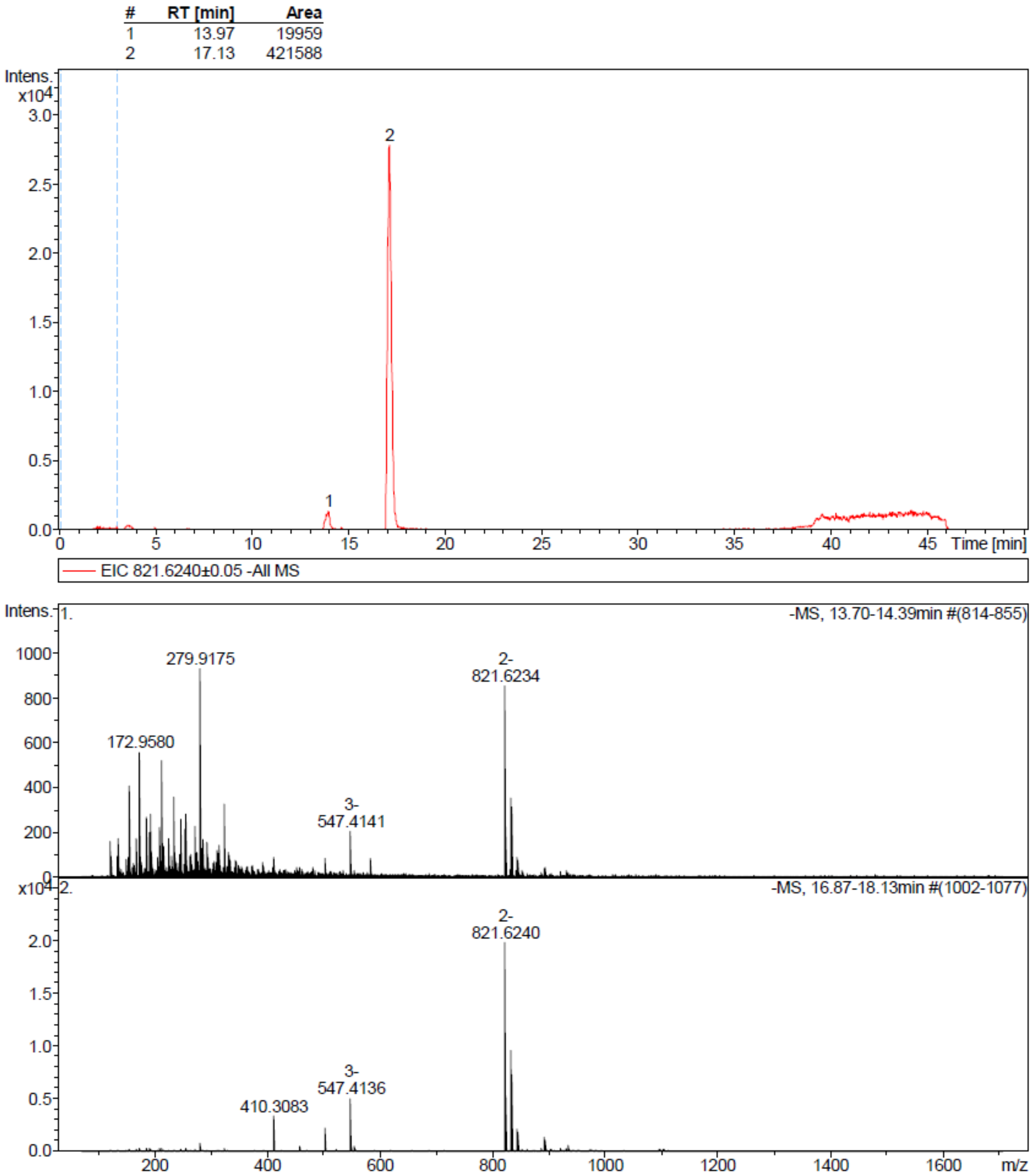

Figure S15: cA$_5$ LC-MS data. Extracted Ion Chromatograms (EIC) obtained by LC-MS for 50μM monodisperse cA$_5$ for calibration (Methods). The main species represented for cA$_5$ is the [M-2H]2-, with a theoretical m/z value of 821.6240, which agrees with the experimental values. The [M-3H]3-(m/z 547.4136) and [M-4H]4- (m/z of 410.3083) species are also observed, although in lower proportion. The pentamer elutes mainly at 17.13 min, with trace amounts eluting at 13.97. The two peaks were taken into account for the calibration, as the pentamer is clearly present in both. The blue dashed line shows the latest elution time for most of the salts and buffer components present in the sample. The calibration was measured in duplicate, right before the validation mixtures, the type III-A and type III-B samples.

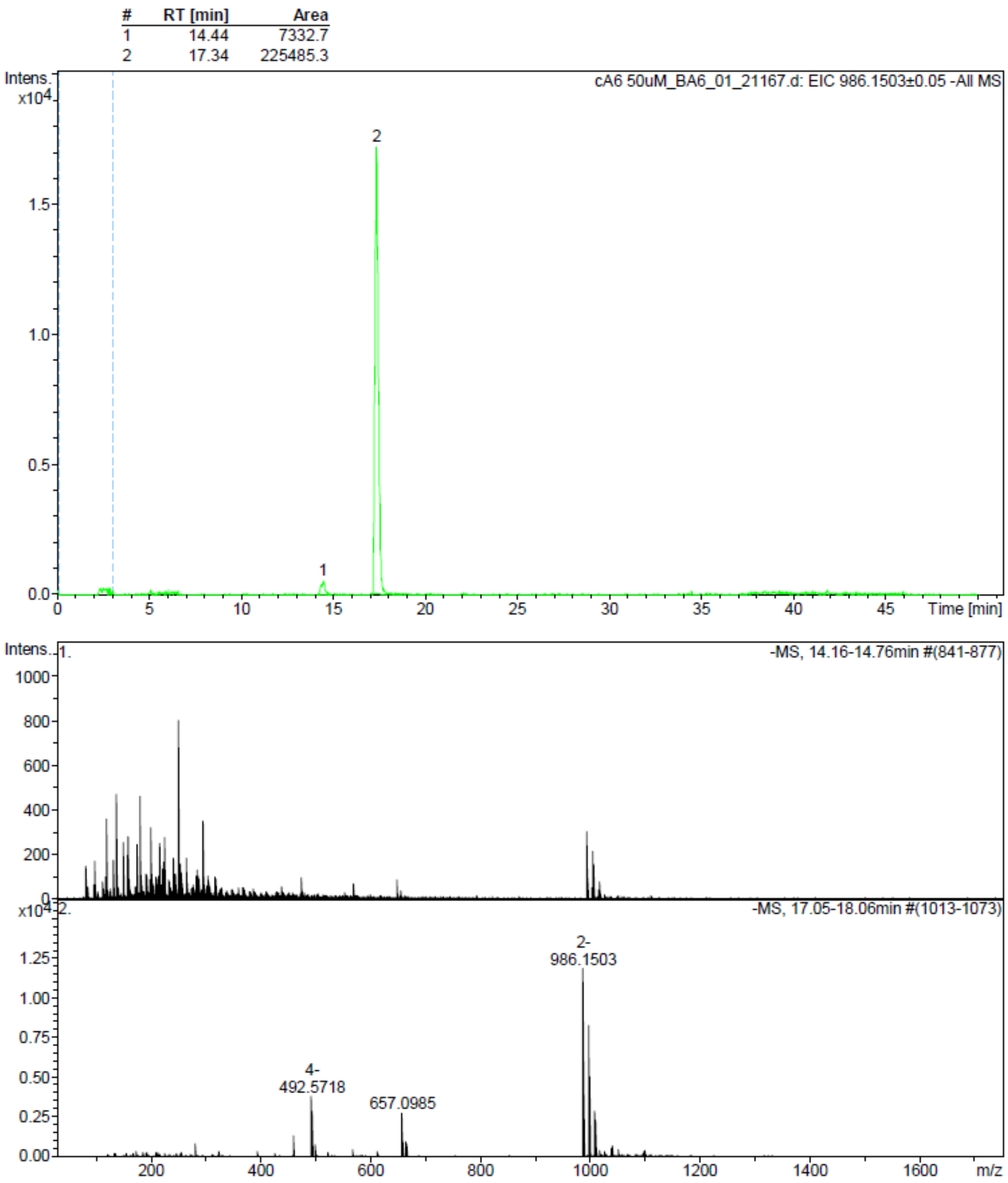

Figure S16: cA$_6$ LC-MS data. Extracted Ion Chromatograms (EIC) obtained by LC-MS for 50µM monodisperse cA$_6$ for calibration (Methods). The main species represented for cA$_6$ is the [M-2H]2-, with a theoretical m/z value of 986.1503, which agrees with the experimental values. The [M-3H]3- (m/z 657.0985) and [M-4]4- (m/z of 492.5718) species are also observed, although in lower proportion. Interestingly, these m/z values for the hexamer overlap with the m/z for the cA$_4$ M-2 species and the cA$_3$ M-2 species, respectively (Figs S11 and S12) giving rise to artifacts in the EIC when the cOAs are present in a mixture together (Figure S15). The hexamer elutes mainly at 17.34 min, with trace amounts eluting at 14.44. The two peaks were taken into account for the calibration, as the hexamer appears in both. The blue dashed line shows the latest elution time for most of the salts and buffer components present in the sample. The calibration was measured in duplicate, right before the validation mixtures, the type III-A and type III-B samples.

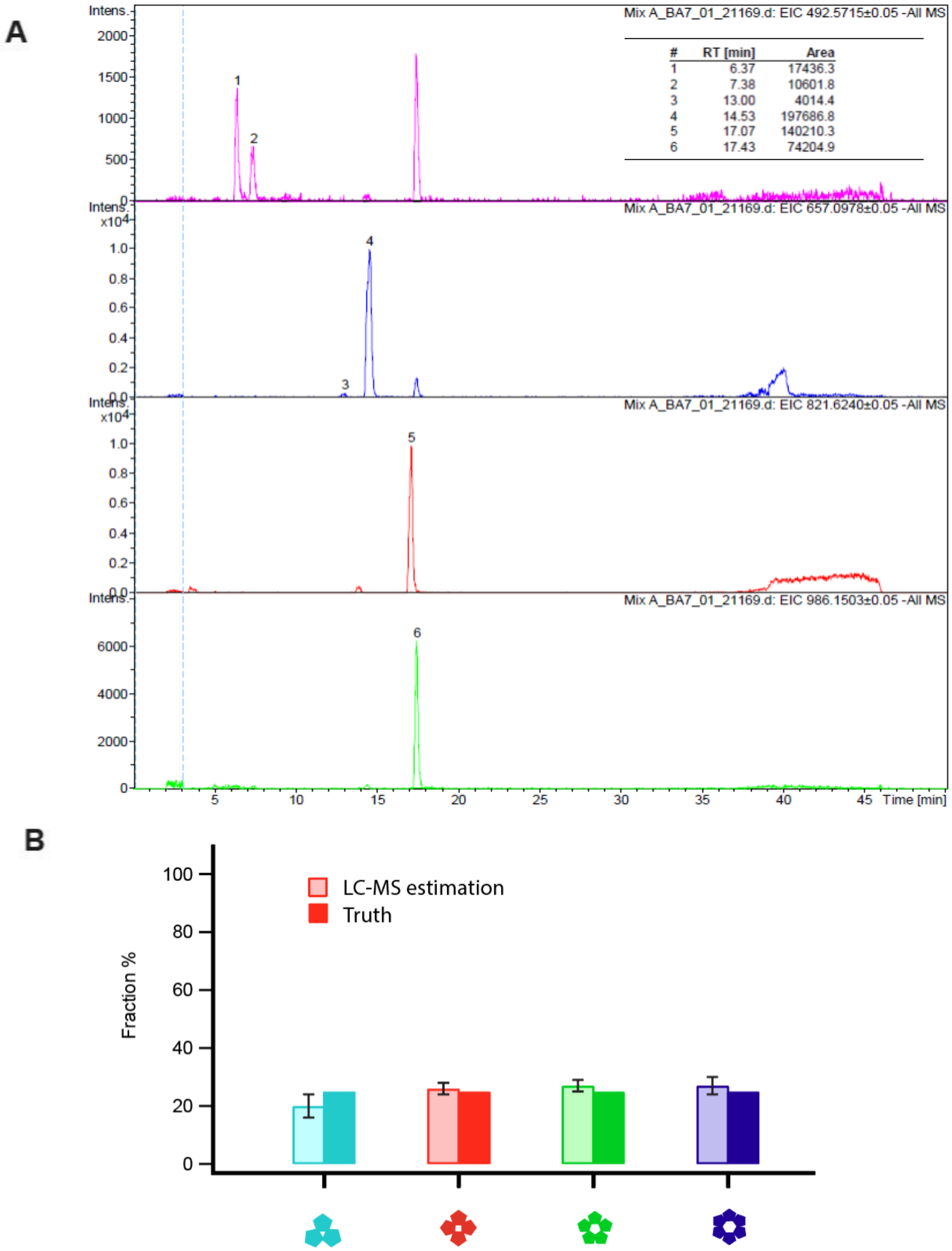

#	RT [min]	Area
1	6.37	17436.3
2	7.38	10601.8
3	13.00	4014.4
4	14.53	197686.8
5	17.07	140210.3
6	17.43	74204.9

Figure S17: Validation of the cOA analysis by LC-MS. A) EICs for the four cOAs (from top to bottom: cA_3, cA_4, cA_5 and cA_6). The peak integrals were used to estimate the cOA ratios (Methods). The EICs for the trimer and tetramer show a peak at 17.43 min, which coincides with the elution time for the hexamer. This is an artifact caused by an overlap of the m/z values between the [M-2H]2- species of the timer and the [M-4H]4- species of the hexamer, and between the [M-2H]2- of the tetramer and the [M-3H]3- of the hexamer (Figure S15), as the trimer and tetramer clearly elute earlier, as seen in Figures S12 and S13. Thus, such peaks were excluded for cA_3 and cA_4 estimation in the mixture. The blue dashed line shows the latest elution time for most of the salts and buffer components present in the sample. The measurements were performed in triplicate. **B)** LC-MS estimation (light colours) vs true values (dark colours) of the validation mixture.

#	RT [min]	Area
1	6.33	2728.8
2	14.48	384258.8
3	16.97	2524.6

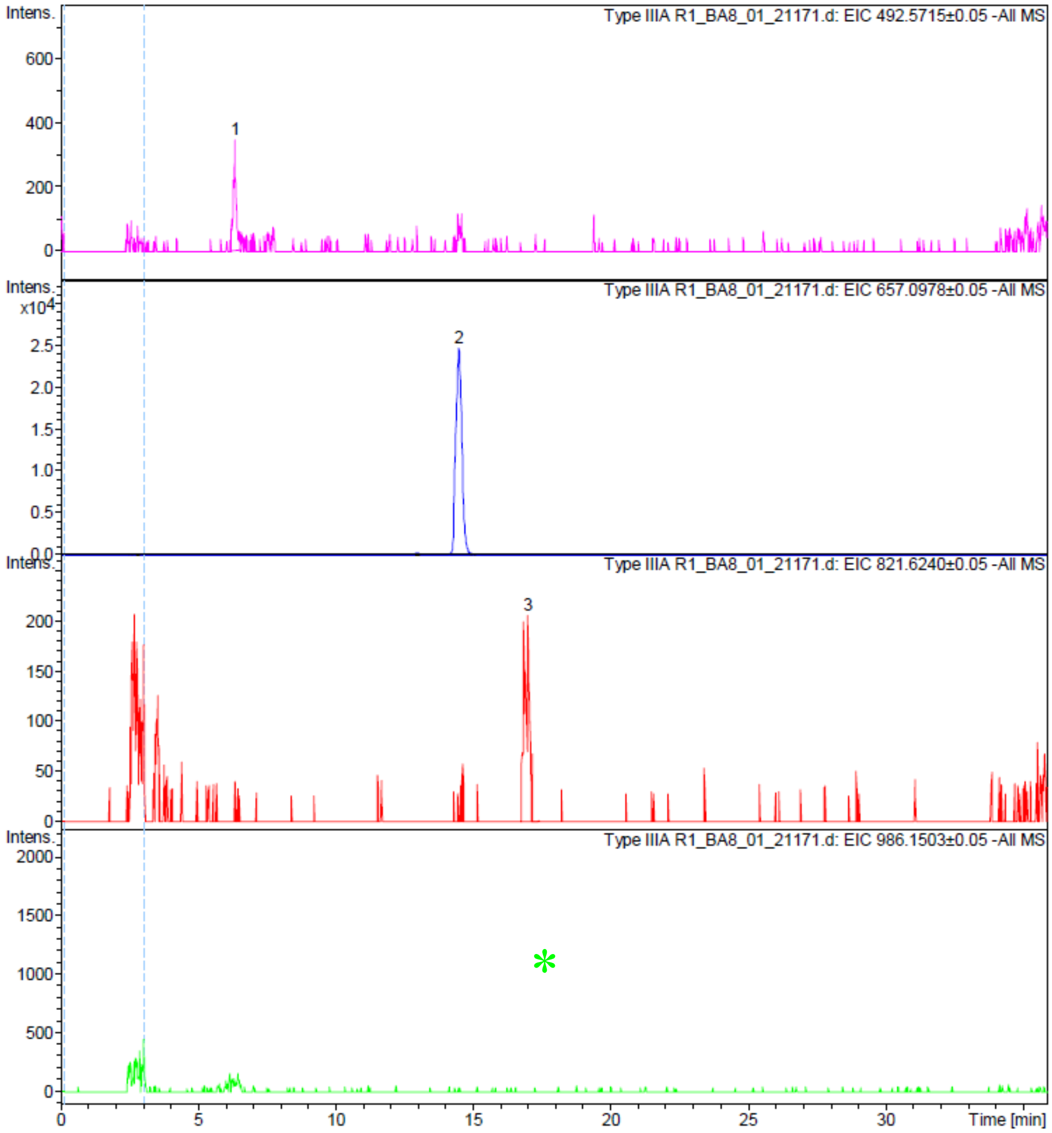

Figure S18: LC-MS results of the cOA produced enzymatically by CRISPR-Cas type III-A: cA_4 is the main component, with only traces of cA_3 and cA_5. No cA_6 is detected. The green asterisk specifies the expected elution time of cA_6. The blue dashed line shows the latest elution time for most of the salts and buffer components present in the sample. The measurements were performed in triplicate.

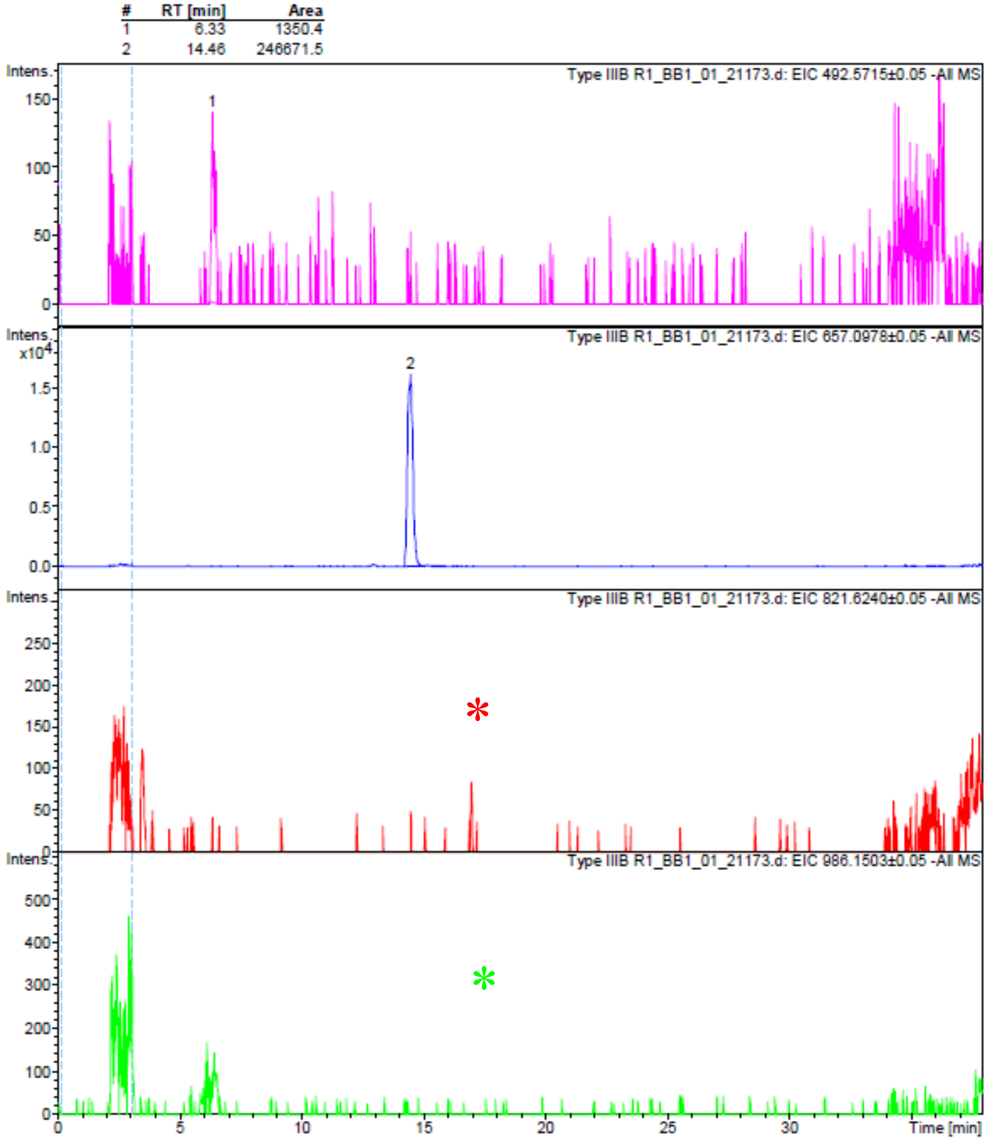

Figure S19: LC-MS results of the cOA produced enzymatically by CRISPR-Cas type III-B: cA_4 is the main component, with only traces of cA_3 and cA_5. cA_6 is not detected in the sample. The red and green asterisks show expected elution time for cA_5 and cA_6, respectively. The blue dashed line shows the latest elution time for most of the salts and buffer components present in the sample. The measurements were performed in triplicate.

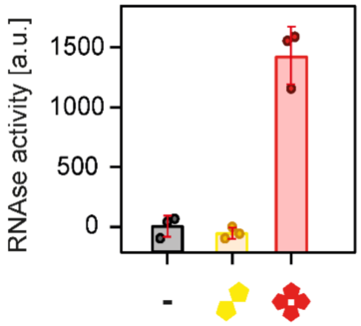

Figure S20: cA_2 does not activate TtCsx1. The RNA cleavage assay (cf. Fig. 5) was extended to cA_2. In contrast to cA_4, it does not activate the RNAse activity TtCsx1 beyond the negative control (-).

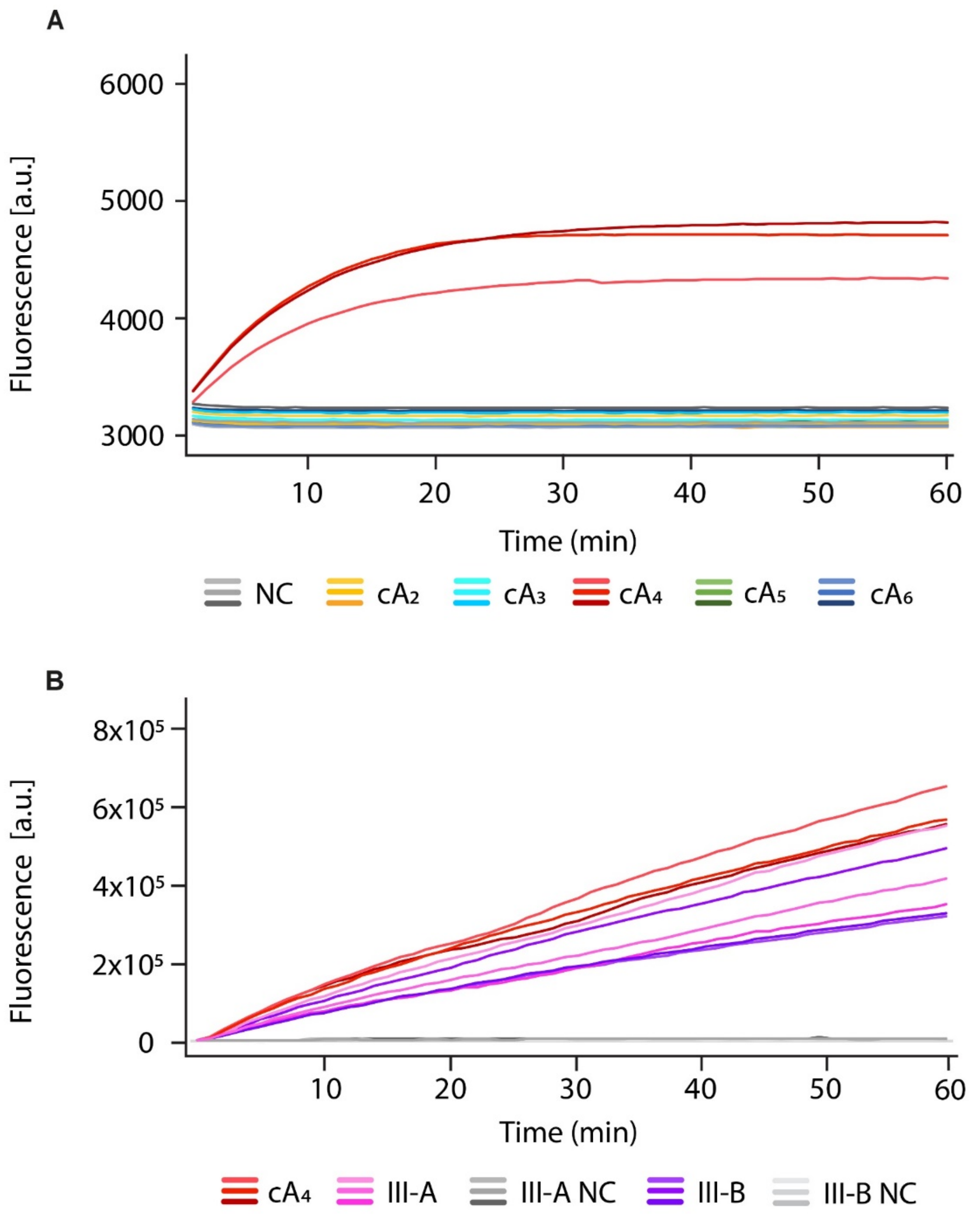

Figure S21: Time-dependent fluorescence data of the CARF activity assays: A),B) Fluorescence values recorded at 1 min intervals in triplicate for the CARF TtCsx1 activations and stringency assay, respectively (see Fig 5A and 5B, Methods). In A), only the incubation with cA_4 activatesTtCsx1, leading to increased fluorescence over time. In B), the incubation of TtCsx1 with cOAs produced by either type III-A and III-B systems leads to an increase in fluorescence indicating cA_4 presence, while there is no increase in fluorescence for the negative controls. cA_4 is used as a positive control for the activation of TtCsx1. A) and B) were measured with different instruments (methods), hence the absolute values (a.u. values) are not comparable between the two.

Table S1: Nanopore event durations of cA_{3-6} at different voltages. The time constants and standard deviations (SD) were obtained by 1,000-fold bootstrapping with replacement, fitting the bootstrapped event duration histograms to an exponential function. The average tau and SD values from the 1,000 bootstrapped fits are reported. The nanopore recordings were performed as described in Figure S2, and the values were calculated from at least n=150 events. All cOAs show the same trend: decreasing event durations with increasing voltages, indicating translocations instead of trapping events. For the smaller cA_3 and cA_4, with shorter event durations, higher voltages push the event durations below the time resolution (25µs) and the events are not resolved. Altogether, a voltage of +120mV was found to provide the optimal balance between distinct event durations, capture rate, and signal-to-noise for the detection of all four cOAs tested in this work.

	Event duration & SD (µs)			
	cA3	cA4	cA5	cA6
+80mV	32±5	109±22	297 ± 33	2010 ± 944
+120mV	15±1	22± 2	96 ± 4	1213 ± 119
+160mV	Not resolved	14±3	61 ± 6	396 ± 39
+200mV	Not resolved	Not resolved	55 ± 5	315 ± 26

Table S2: Pair-wise statistical difference and equivalence test p-values, for neural network classifications of cOA mixtures. t-test p-values and two one-sided t-test (TOST) procedure p-values are reported for difference and equivalence testing respectively. Nomenclature: sample '50_40_10' indicates 50% $cA_{3/4}$, 40% cA_5 and 10% cA_6. While the cA_5 and cA_6 in the 80_10_10 mixture differ significantly according to the t-test ($p << 0.01$), in pairwise equivalence tests, they deviate significantly less than 10% (TOST procedure, $p<0.05$). The cA_5 and cA_6 fractions differ less than 10% from each other for both samples (TOST procedure, $p<<0.01$).

Sample:	cA_A	cA_B	p_{TOST}	$p_{t\text{-}test}$
50_25_25	$cA_{3/4}$	cA_5	1	3.77×10^{-13}
50_25_25	$cA_{3/4}$	cA_6	1	2.71×10^{-13}
50_25_25	cA_5	cA_6	1.21×10^{-5}	4.88×10^{-1}
50_40_10	$cA_{3/4}$	cA_5	1	1.95×10^{-16}
50_40_10	$cA_{3/4}$	cA_6	1	2.61×10^{-19}
50_40_10	cA_5	cA_6	8.18×10^{-1}	1.25×10^{-8}
80_10_10	$cA_{3/4}$	cA_5	1	3.76×10^{-20}
80_10_10	$cA_{3/4}$	cA_6	1	8.54×10^{-21}
80_10_10	cA_5	cA_6	3.76×10^{-2}	1.33×10^{-6}
type III-A	$cA_{3/4}$	cA_5	1	1.29×10^{-305}
type III-A	$cA_{3/4}$	cA_6	1	1.44×10^{-300}
type III-A	cA_5	cA_6	3.33×10^{-91}	2.07×10^{-74}
type III-B	$cA_{3/4}$	cA_5	1	6.03×10^{-269}
type III-B	$cA_{3/4}$	cA_6	1	4.25×10^{-261}
type III-B	cA_5	cA_6	3.56×10^{-66}	2.65×10^{-50}

Table S3: Multi-step gradient used during LC-MS measurements. For the separation of the different cOAs during liquid chromatography, a multi-step gradient of acetonitrile (mobile phase B) was applied, as indicated in methods.

time in min	mobile phase B in %
0	2
5	2
28	15
30	50
32	50
34	95
41	95
42	2
50	2
